# PoolSeq Genome‐Wide Association Studies and Microbial Signature Analyses Identify Novel Candidates Associated With Pyrethroid Resistance Evolution in *Anopheles funestus* in Cameroon

**DOI:** 10.1111/mec.70220

**Published:** 2026-01-05

**Authors:** Mahamat Gadji, Jonas A. Kengne‐Ouafo, Magellan Tchouakui, Murielle J. Wondji, Leon M. J. Mugenzi, Jack Hearn, Boyomo Onana, Charles S. Wondji

**Affiliations:** ^1^ Centre for Research in Infectious Diseases (CRID) Yaoundé Cameroon; ^2^ The University of Yaoundé 1 Yaoundé Cameroon; ^3^ Liverpool School of Tropical Medicine Pembroke Place Liverpool Liverpool UK; ^4^ Syngenta Crop Protection, Werk Stein, Schaffhauserstrasse Stein Switzerland; ^5^ Centre for Epidemiology and Planetary Health Scotland's Rural College (SRUC), RAVIC Inverness UK

**Keywords:** *Anopheles funestus*, *Elizabethkingia anophelis*, genomic, malaria, PoolSeq

## Abstract

Intensification of insecticide resistance in malaria vectors is undermining efforts to sustain control strategies. The evolutionary features underlying such exacerbation in major vector such as *Anopheles funestus* are only partially understood. PoolSeq whole genome analysis of *Anopheles funestus* from Mibellon (Cameroon), (alive and dead at 1×, 5× and 10× concentrations), failed to identify hits associated with resistance escalation. However, stronger signals emerge at the *rp1* and *CYP9* loci when comparing these phenotypes to the susceptible reference strain FANG, with genomic analysis using F_3_ crosses implicating these regions in resistance evolution. Temporal genomic between 2014 and control unexposed 2021 populations confirmed substantial genomic changes largely confined to these two regions with evidence of selective sweeps linked to the presence of multiple novel replacement polymorphisms and signatures of complex genomic evolution emerging from major cytochrome P450 genes within the *CYP9* and *rp1* regions at increasing allelic frequencies in field individuals and alive genetic crosses, indicating that those variants are potentially driving resistance evolution. Combined genotyping of the *rp1*‐based 4.3 kb SV and *CYP9K1* (G454A) in alive and dead genetic crosses underscores their significant contribution to super‐resistant phenotype in *Anopheles funestus* population in Mibellon. On the other hand, microbial composition changes, notably *Elizabethkingia anophelis* was associated with resistance evolution, suggesting their potential role in shaping the resistance phenotype while *Serratia marcescens* and *Asaia bongorensis* correlate with susceptibility. Genetic events and microbial symbionts associated with resistance evolution offer promising avenues for developing molecular markers to manage insecticide resistance.

## 1 Introduction

The burden of malaria is still high in Africa, and particularly in sub‐Saharan Africa, which accounts for about 95% and 96% of the global burden (cases and deaths, respectively) of this disease despite the promising control intervention strategies (WHO 2024). Unfortunately, those who pay the heaviest price to this disease are pregnant women and children under the age of 5 with nearly half a million African children dying every year (Perin et al. 2022).

Cameroon is highly endemic and part of high‐burden, high‐impact countries (HBHI) (WHO‐HBHI 2020). It accounts for approximately 6.46 million cases and 9000 deaths in 2022 (WHO 2023). Malaria has significant economic and health impacts in Cameroon (Tchicaya et al. 2014), with the northern regions being the most affected, accounting for a substantial portion of healthcare consultations (30%) and hospitalisations (64%) mainly in children under 5 years (NMCP 2021).

The current malaria prevention toolbox includes chemoprophylaxis, chemotherapies, vaccines recently recommended (RST, S/AS01 and R21/Matrix‐M) and rollout in Cameroon (Ndoula et al. 2024) and importantly vector control tools (VCTs) through insecticide treated nets (ITNs) and indoor residual spraying (IRS) which have shown to have significantly lessen malaria burden between 2000 and 2015 in sub‐Saharan Africa with the greatest part of the reduction attributed to Long Lasting Insecticidal Nets (LLINs) (Bhatt et al. 2015).

Despite the progress made by governments and the collaborative efforts of Malaria Control Programmes, and their partners in different countries and particularly in Cameroon to advance towards malaria elimination, several factors are limiting the goal of eliminating malaria by 2030. King among these is insecticide resistance escalation due to the widespread of VCTs coupled with pesticides used in agriculture and environmental pollution by human activities, that has led to the adaptation and the tolerance of insecticide by major malaria vectors notably *Anopheles gambiae* and *Anopheles funestus*. This is why these vectors are now able to survive exposure to the diagnostic doses of insecticide recommended by the WHO. Worryingly, some of them are even surviving 5 and 10 times (5× and 10×) the diagnostic concentration of these insecticides mainly the pyrethroids (type I and II) which are the major active ingredient broadly recommended for impregnation of bed nets because of their high performance and very low toxicity to humans (Mosha et al. 2022). Insecticide resistance evolution pattern is heterogenous across Africa and several African regions have reported resistance escalation to nearly all classes of insecticides for a wide range of species with recurrent and consistent reports from Nigeria, Mozambique, Malawi, Uganda, Ghana, Democratic Republic of the Congo (DRC), Chad and Cameroon (Menze et al. 2022; Mugenzi et al. 2022; Nguiffo‐Nguete et al. 2023; Tchouakui et al. 2021).

Resistance is mediated by various mechanisms namely target site insensibility (Grigoraki et al. 2023) known as knock down resistance mainly evidenced in *Anopheles gambiae* sl and recently, in *Anopheles funestus* restricted just to DDT in Tanzania (Odero et al. 2024), behavioural resistance (Kreppel et al. 2020), cuticular penetration resistance (Jacobs et al. 2023), recently microbial mediated pathway (Dada et al. 2018), and metabolic resistance mediated by the expression of metabolic enzymes able to metabolise or sequestrate the active ingredient before it reaches its target site (Ingham et al. 2023; Ramkumar et al. 2023).

Several studies have linked the resistant mechanism in *Anopheles funestus* to the overexpression of metabolic enzymes such as the major enzyme superfamilies of cytochrome P450 monooxygenases and the Glutathione S transferase gene clusters. Thus, some of these genes have been subsequently validated as pyrethroids metabolisers (Ramkumar et al. 2023; Tatchou‐Nebangwa et al. 2024; Djoko Tagne et al. 2024) and molecular markers to monitor their spread are available (Tatchou‐Nebangwa et al. 2024; Djoko Tagne et al. 2024; Mugenzi, Tekoh, Ntadoun, et al. 2024). The high diversity, the complexity of xenobiotic biodegradation pathways and the high resistance intensity on the field make the identification of new candidates driving resistance escalation challenging. The methods used to detect candidates so far have been effective in identifying metabolic resistance candidate genes acting through overexpression, but they failed to pinpoint the genomic variations associated to their overexpression or any other pathway linked to insecticide resistance escalation.

Thus, our study employed a comprehensive MultiOmics approach, evaluating the resistance profile to pyrethroids at 1×, 5× and 10× concentrations then performing comparative genomic between each condition to detect signals associated with high resistance intensity. Subsequently, we generated hybrid populations through genetic crosses of resistant and susceptible populations. Furthermore, we carried out temporal genomic analysis using populations collected before and after vector control interventions that is, 2014 and 2021 to validate the signals. We also profiled microbial signatures under each condition, identifying microbial symbionts associated with resistance or susceptibility. Lastly, we assessed the combined impact of two molecular markers located within the major signals, on the resistance evolution using genetic crosses.

## 2 Materials and Methods

### 2.1 Mosquito Collection Site, Rearing and Susceptibility Profile

Adult F_0_ *Anopheles* mosquitoes were collected in Mibellon (6°46′ N, 11°70′ E), a village in Cameroon located in the Adamawa region, Mayo Banyo Division and Bankim Sub‐division (Figure S1). It is a mountainous area and forms the barrier between Cameroon's forested south and savanna north. It is located in close proximity to permanent water bodies, including a lake and swamps which provide suitable breeding sites for mosquitoes. Human activities are mainly fishing, hunting and subsistence farming, including maize, watermelon, tomatoes and coffee plantations. *An. funestus* s.s. was the main malaria vector in the area and were highly resistant to pyrethroid and DDT (Tazokong et al. 2024; Menze et al. 2018). *Plasmodium falciparum* is the major parasite ensuring the transmission of malaria in Mibellon and was associated to high *Plasmodium* sporozoite infection (Menze et al. 2018; Tchouakui et al. 2019). A survey in the area revealed a high usage of pyrethroids, neonicotinoids and carbamates insecticides in the coffee and watermelon farms. Wild caught *An. funestus* mosquitoes blood fed and resting indoors were collected using an electric aspirator between November 2020 and 2021 in Mibellon. These mosquitoes were forced to lay eggs post 5 to 6 days of collection (Morgan et al. 2010), reared at the Centre for Research in Infectious Diseases (CRID) insectary till F_1_ adults. Three to five days unfed adult's female were exposed to all the four classes of insecticides including pyrethroid type I and type II at increasing concentrations (1×, 5× and 10×) according to WHO guideline to access the resistance profile as well as the resistance intensity and to select alive and dead mosquitoes for Genome‐wide association study (GWAS). Additionally, due to high level of resistance observed post insecticide bioassay, reciprocal genetic crosses were done between FANG which is the fully susceptible strain originated from Angola and Mibellon which is the multiple insecticide resistant strain, and exposed at F_2_ generation using time exposure to select highly susceptible at 15 min and highly resistant at 1 h. All those samples were stored either in ethanol 80% for the alive or in silica gel for the dead in order to carry out temporal genomic analysis. Some F_1_ unexposed samples were also stored in order to carried out temporal genomic analysis with samples collected in 2014 in the same locality (Weedall et al. 2020a).

### 2.2 Establishment of Genetic Crosses

**FIGURE 1:**
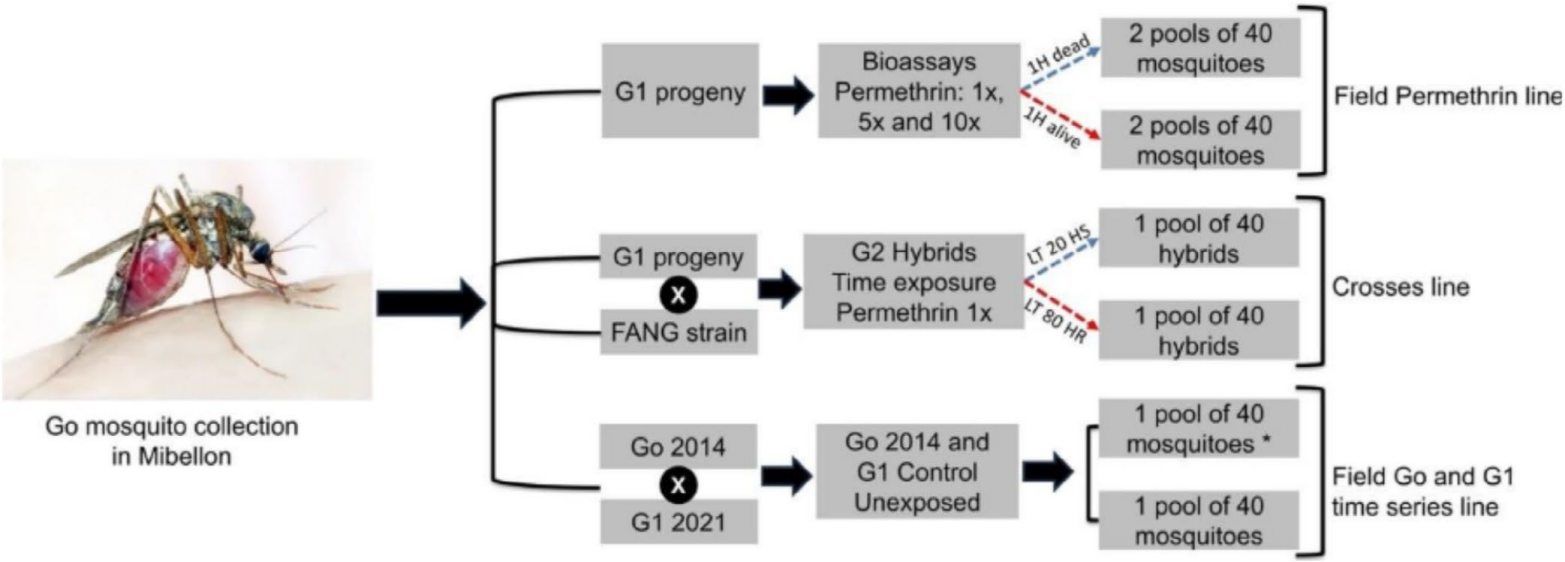
Experimental design flowchart. LT_20_ and LT_80_ are the lethal time required to kill 20% and 80% of the mosquito population, respectively. HR and HS mean highly resistant and highly susceptible, respectively. * indicates that the sample was used in previous publication.

Reciprocal genetic crosses were established at the Centre for Research in Infectious Diseases (CRID) insectary using the FANG fully susceptible strain, which was originally colonised in Angola, and the Mibellon multiple insecticide resistant *Anopheles funestus* s.s. strain from Adamaoua, Cameroon (Figure 1). The purpose of these genetic crosses was to break the genetic linkage and determine the specific genotypes associated with the observed phenotypes because the high resistance intensity in the field hindered the ability to segregate the genotypes and assess the effective role of resistant alleles on mosquito phenotypes.

To carry out the reciprocal crosses, pupae from the FANG and Mibellon strains were individually placed in Falcon tubes to allow them to emerge as adults. Female mosquitoes that emerged were separated from males. Reciprocal crossing was carried out with approximately 150 mosquitoes from Mibellon and 150 from FANG in two different cages (30 × 30 cm). These crosses were allowed about 7 days to maximise mating rate, and the mosquitoes were blood‐fed and supplement with 10% sucrose solution. The F_1_ individuals were allowed to reproduce freely and blood‐fed again to generate the F_2_ and F_3_ generations under standard insectary conditions. The larvae were fed with Tetramine baby fish food. The hybrid crosses from field males and females from FANG were successful in rearing and were then segregated by exposing 3 to 5‐day‐old unfed females to time exposure with WHO bioassays. Highly susceptible (HS) phenotypes were selected at 12 min to permethrin, which corresponded to the time required to kill approximately 20% of the mosquitoes (LT_20_). Highly resistant (HR) phenotypes were selected at 1 h, corresponding to the time required to kill approximately 80% of the mosquitoes (LT_80_). The HR and HS hybrid phenotypes were used for subsequent GWAS and combined genotyping of resistant markers.

### 2.3 Genomic DNA Extraction, Pooling, Library Preparation and Pool Template Whole Genome Sequencing

For the PoolSeq GWAS experiment, there were two technical replicates (one pool for alive and dead 5×) consisting of 40 individuals per pool, per phenotype and per level of concentration (1×, 5× and 10×) as well as for control unexposed samples. Additionally, there were two replicates available for the genetic crosses, one consisting of 40 individuals classified as highly susceptible which was selected using time exposure with permethrin 1× at 15 min (LT_20_) and the other consisting of 40 individuals classified as highly resistant selected at 1 h (LT_80_). In addition to the replicates mentioned above, F_1_ unexposed female samples were also available for PoolSeq GWAS temporal analysis. Genomic DNA was extracted from each individual using the DNeasy Blood and Tissue kit from Qiagen (Qiagen 2020). Molecular species identification was performed using a method described by (Koekemoer et al. 2002). The gDNA samples were pooled in set of 40 individuals per replicate, per phenotypes (alive or dead), and concentrations (1×, 5× and 10×), in equal amounts of gDNA. Two control pools containing water and ethanol were also included to serve for microbial analysis. Prior to pooling, the gDNA samples were purified using Rnase A and quantified using a Qubit instrument. Library preparation, quality control and whole‐genome sequencing with Novaseq 6000 (2 × 150 bp paired‐end) was carried out by Novogene company, University of Cambridge, UK using Illumina platform principle based on sequencing by synthesis.

## 3 Bioinformatic and Statistical Analyses

### 3.1 Quality Control of Reads, Alignment and Filtering

The whole pipeline and scripts with the details of parameters used for the PoolSeq analysis are available on GitHub via https://github.com/Gadji‐M/PoolSeq_OMIcsTouch. The quality control of the raw fastq files obtained from Novogene was performed using FastQC (https://www.bioinformatics.babraham.ac.uk/projects/fastqc/) and the outputs were piped into Multiqc to aggregate and visualise the final results (Ewels et al. 2016). Reads were trimmed based on QC outputs with Trimmomatic (Bolger et al. 2014) based on the following parameters: LEADING:10 TRAILING:10 SLIDINGWINDOW:4:10 MINLEN:50. For the alignment step, the reference genome of *Anopheles funestus* (VectorBase‐61_AfunestusFUMOZ_Genome.fasta) was downloaded from VectorBase and indexed (https://vectorbase.org/vectorbase/app/downloads/release‐61/AfunestusFUMOZ/fasta/data/). The ‘bwa mem’ (version 0.s7.17) command line was executed on Ubuntu/Linux terminal with default parameters. The output was then piped using the ‘samtools view ‐Sb ‐’ (version 1.13) command line to generate a bam file containing only reads with a quality phredscore greater than 10 (−q 10). The output bam alignment files from all pools were processed using the PICARD tool https://broadinstitute.github.io/picard/ for coordinate sorting and duplicate marking. The “java ‐jar picard.jar SortSam” command was used to sort the input bam files. For duplicate marking, the “java ‐jar picard.jar MarkDuplicates” command with the “REMOVE_DUPLICATES false” option was employed. This step identified and tagged duplicate reads in the sorted bam files, with duplicates defined as reads originating from the same gDNA fragment. Additionally, a metrics file was generated to track the number of duplicates for paired‐end reads.

### 3.2 Creation of Mpileup and Synchronised Files for Downstream Analyses

Synchronised files serve as the primary input files for PoPoolation2 and contain allele frequencies for each population (pool) at every base in the reference genome in a compact format. To generate a synchronised file, a multiple pileup file (mpileup) was first created, which combines data from all the population pools. The “Samtools mpileup ‐B ‐Q 0” command was used on the bam files to generate a single mpileup file encompassing all the pools. This mpileup file was then processed using the “java ‐ea ‐jar mpileup2sync.jar” command in popoolation2 to generate the synchronised output file (Kofler, Pandey, and Schlötterer 2011).

### 3.3 Population Genomic Analysis for Detection of Signature of Selective Sweeps Associated to Increase Pyrethroid Resistance in *Anopheles funestus* in Mibellon

Custom shell script (Fst_sliding_windows.sh) based on Popoolation2 (Kofler, Pandey, and Schlötterer 2011) was used to compute the exact allele frequency and the pairwise *F*_*ST*_ genetic differentiation between phenotypes. The exact allele frequency differences between populations were calculated using “perl snp‐frequency.pl” from popoolation2 with the synchronised file as input. Parameters to run the script were ‐‐min‐count 2, ‐‐min‐coverage 10 and ‐‐max‐coverage 95%. This script generates two outputs files having two different extensions. The file containing the differences in allele frequencies for every pairwise comparison of the phenotypes (i.e., having this extension *_pwc) was used to compute principal component analysis (PCA) for phenotype structure assessment. Pairwise average *F*_*ST*_ was computed using the custom script Fst_sliding_windows.sh for various overlapping sliding windows ranging from 50,000 to 500,000 bp. To validate the above outputs, pairwise *F*_*ST*_ was also calculated for each SNPs using popoolation2 and poolfstat then windowscanr (https://github.com/tavareshugo/WindowScanR) was utilised for windows analyses. Parameters used to run the script are found in GitHub via https://github.com/Gadji‐M/PoolSeq_OMIcsTouch. Additionally, *F*_*ST*_ files were formatted and relevant comparisons were visualised in R via ggplot2 package (Wickham and Wickham 2016).

To show evidence of selective sweeps among our populations/phenotypes, Genome‐wide Tajima's D values and nucleotide diversity (pi) measures were computed for each sample then average Tajima's D and pi were computed for combined replicates/phenotypes. Mpileup files were generated and subsampled to uniform coverage before computing the measures. The “samtools mpileup” command was used to generate the mpileup files, which were then subsampled (with replacement) to a uniform coverage depth of 20× using the “subsample‐pileup.pl” perl script from popoolation1 (Kofler, Orozco‐terWengel, et al. 2011). Sites with coverage depths less than 10 or greater than the 95th centile of coverage depth were removed. The resulting files were utilised to estimate Tajima's D and pi in overlapping windows of 50 kb moving in step sizes of 25 kb. Grenedalf https://github.com/lczech/grenedalf was used to confirm the outputs generated by popoolation2 (Kofler, Pandey, and Schlötterer 2011). To identify potential selective sweeps, fine scale resolution was employed by zooming into major peaks genome‐wide in overlapping windows of 1 kb moving in steps of 0.5 kb.

### 3.4 Variant Calling and Filtering for the Detection of SNPs and Short Indels

Variant calling was done with VarScan which employs heuristic and statistic thresholds based on user‐defined criteria to call variants using Samtools mpileup data as input to detect SNPs/indels in individual and pooled samples (Koboldt et al. 2009). Outputs obtained post VarScan were annotated and variants were predicted using SnpEff version 5.1. Indeed, SnpEff is a variant annotation and effect prediction tool (Cingolani et al. 2012). It annotates and predicts the effects of genetic variants (such as amino acid changes). This tool tells us on to which genes we should focus further analyses. *Anopheles funestus* database was build and SnpEff was run using the command line java ‐Xmx8g ‐jar snpEff.jar and the output vcf annotated file was filtered with SnpSift and bcftools (Danecek et al. 2021).

### 3.5 Analysis of Signature of Complex Genomic Evolution

For a comprehensive exploration of the signature of complex genomic evolution in *Anopheles funestus* genome, a combination of computational tools and Integrative Genomic Viewer (IGV)‐based analysis and visualisations of alignment files were employed to identify genomic anomalies. INSurVeyor was utilised for detecting large structural variations (SVs) such as insertions, and deletions (indels) (Rajaby et al. 2023). Concurrently, Smoove, available at https://github.com/brentp/smoove, was employed to identify duplications, inversions, and large indels involving the execution of the “java ‐jar picard.jar FixMateInformation” command on each BAM file to include mate cigar information in each read and population. Subsequent to mate information adjustment, SV calling was executed individually for each population using the “python insurveyor.py” command with ‐‐threads set to 40 for efficient parallel processing. The resulting vcf files were merged using SurVClusterer (https://github.com/Mesh89/SurVClusterer). Additionally, SV calling was conducted in Smoove through conda, utilising the “smoove call” command and the identified variants were annotated using “smoove annotate” command. All the SVs detected were filtered with “bcftools filter” command (Danecek et al. 2021).

The findings obtained through computational tools were compared using the Integrative Genomic Viewer (IGV) to visually validate the existence of genomic alterations. The inspection of BAM alignment files aimed to infer genomic anomalies based on four key metrics. Firstly, increased coverage depth in specific gene clusters or genomic regions, indicating duplication with the presence of more than one copy of the genomic region or a large DNA fragment insertion. Secondly, the identification of read pairs with incorrect insert sizes within the genomic regions. Thirdly, the detection of abnormal relative read pair orientations (Figure S2) suggesting a range of structural variations, including duplication, intra or inter chromosomal translocation, indels, and chromosomal inversions. Lastly, the observation of multiple seemingly chimeric and discordant reads spanning putative breakpoints, either clipped thereafter or not, in the alignment, further contributed to the inference of genomic alterations.

### 3.6 Polymorphism Analysis of *CYP6P9b* Gene and Combined Impact of 4.3 Kb Structural Variant and *CYP9K1*‐*G454A* on Super‐Resistance in *Anopheles funestus* in Mibellon

Attention was given to the *CYP6P9b* gene following the post‐PoolSeq analysis due to its notable pattern of genetic variation. Two major SNPs (V392F and V359I) were identified at very low frequencies in the 2014 population, became fixed by 2021, and apparently segregating between alive and dead in genetic crosse phenotypes. Additionally, the absence of these SNPs in FANG highly susceptible strain prompted us to leverage Sanger sequencing to perform a phenotype–genotype association study. To achieve this, primers (Table S1) were design to amplify the full length of *CYP6P9b* gene in 10 permethrin‐highly resistant and 10 highly susceptible hybrids. PCR products were purified using the ExoSAP protocol and directly sent for Sanger sequencing. Fasta sequences were received after Sanger sequencing then aligned in multiple alignments using ClustalW from BioEdit software (Hall 1999). Population genetic parameters, including nucleotide diversity and haplotype diversity were assessed using the full coding sequence of *CYP6P9b* in DnaSP version 6.12.03 (Rozas et al. 2017). Haplotype network was built using the TCS program (Múrias dos Santos et al. 2016) and a maximum likelihood phylogenetic tree was constructed using MEGA 11 (Tamura et al. 2021).

Previous studies have shown that 4.3 kb SV (Mugenzi, Tekoh, Ntadoun, et al. 2024) is associated to pyrethroids resistance in *Anopheles funestus* in Central and East Africa, as well as a point nonsynonymous mutation on the *CYP9K1* (G454A) conferring resistance in East and Central Africa (Djoko Tagne et al. 2024; Hearn et al. 2022), we took advantage of the simple PCR DNA‐based diagnostic tools design in the previous studies (Djoko Tagne et al. 2024; Mugenzi, Tekoh, Ntadoun, et al. 2024) to assess the combined impact of those two genomic variations on super‐resistant phenotype using genetic crosses. The genotyping involved 44 highly resistant and 44 highly susceptible genetic crosses samples. The Polymerase Chain Reaction (PCR) reaction was carried out using 0.51 μL of each 10 mM primer specific to each variant and 1 μL of genomic DNA as template in 15 μL final reactions containing 10× Kapa Taq buffer A, 0.12 μL of 25 mM dNTPs, 0.75 μL of 25 mM MgCl2, 0.12 μL of 1 U Kapa Taq enzyme (Kapa biosystems), 7.515 μL of deionised water and 1.875 μL of Bovine Serum Albumin (BSA). The cycle conditions were: 1 cycle pre‐denaturation at 95°C for 5 min; 30/35 cycles of denaturation at 94°C for 30 s, annealing at 58°C/60°C for 30 s, extension at 72°C for 1 min and then a final extension step at 72°C for 10/5 min, for the *CYP9K1* and 4.3 kb SV, respectively. PCR amplicon products were separated on 1.5% agarose gel by electrophoresis, stained with Midori Green Advance DNA Stain (Nippon Genetics Europe GmbH) and visualised on a UV transilluminator gel documentation. Primer sequences used for the PCR are available in (Djoko Tagne et al. 2024; Mugenzi, Tekoh, Ntadoun, et al. 2024) publications. This method detects homozygote resistant (454A/A‐RR and SV+/SV + ‐RR) at 216 bp and 780 bp for *CYP9K1* and 4.3 kb SV, respectively, homozygote susceptible (G/G 454 ‐SS and SV‐/SV‐‐SS) at 434 bp and 281 bp, respectively and heterozygote (G454A/SV + SV‐‐RS) with both resistant and susceptible bands. Genotype and phenotypes association was assessed by estimating the pairwise odds ratio of resistant phenotype between the homozygous resistant, heterozygote and homozygous susceptible individuals compared to dead phenotype. Fisher statistic was used to access the combined impact of these bi‐variants on resistance evolution.

### 3.7 Metagenomic Assembly and Metataxonomic Classification Among Resistant and Susceptible Phenotypes

To evaluate the role of microbial symbionts in insecticide resistance evolution using Whole Genome Sequencing data, a set of custom Bash scripts was developed. These scripts were designed to extract unmapped reads from all BAM files through the “samtools view ‐f ‐b 4” command. The extracted reads were then converted into FASTQ files using the “bamtofastq” and “seqtk” commands. Subsequently, the latest Kraken2 standard database version 2.1.2 (May 10, 2021) with indexes was cloned (Wood et al. 2019a) and executed via “Kraken2_classification.sh” shell script with 50 threads for each sample pool. Contigs were assembled in megahit (Li et al. 2015) following the shell script ‘assembly_script.sh’ available on GitHub repository via https://github.com/Gadji‐M/PoolSeq_OMIcsTouch. The assembled contigs were then converted into 6‐way open reading frames using TransDecoder.LongOrfs. Microbial classification was done again to confirm the first results in kraken2 database pre‐ and post‐contigs assembly (Wood et al. 2019b). The Kraken2 classification generated four files, among which the Kraken report file of each sample was input into Pavian (Breitwieser and Salzberg 2020). Pavian generated features that were filtered based on abundance values (> 0.01) and the number of reads (> 500) in all samples. Pavian outputs and classified sequences in fasta format were processed for downstream analyses including diversity and differential abundance analysis.

### 3.8 Microbial Community Diversity Analysis

Outputs from pavian were subsequently suggested to removal of features associated with controls (Ethanol and molecular water) using R decontam package and remaining features were processed in downstream analysis (Davis et al. 2018). Alpha (Shannon and Simpson) and beta diversities were analysed in phyloseq and compared between different treatments (concentrations) and phenotypes. Shannon diversity Index takes into account both the number of species (species richness) and their evenness and was calculated using ‘estimate_richness ()’ function in Phyloseq package (McMurdie and Holmes 2013). A pairwise Wilcoxon non parametric tests with *p*‐value adjustment, using the Bonferroni FDR method was used to compare alpha diversity between different treatments and phenotypes. Multivariate analysis was done to compute beta diversity based on Bray Curtis dissimilarity distance and displayed in Principal Component Analysis (PCoA). Output distance matrices were compared between different treatments and phenotypes using permutational multivariate analysis of variance (PERMANOVA) with 999 permutations supplemented with Benjamini‐Hochberg FDR corrections. All the diversity analysis was done in phyloseq package (McMurdie and Holmes 2013) and visualised in R using ggplot2 (Wickham and Wickham 2016).

### 3.9 Differential Abundance Analysis Between Various Treatments

Differential abundance analyses were done using DESeq2 (Love et al. 2014). It takes care of the *p*‐value adjustment based on Benjamini‐Hochberg (BH) FDR to control for multiple testing. DESeq2 utilises a negative binomial distribution to detect differences in read counts between groups. Its normalisation takes care of the differences between library sizes and compositions. DESeq2 analysis includes multiple steps, but they are done automatically. The analysis of composition of microbiomes with bias correction (ANCOM‐bc2) (Lin and Peddada 2020) was employed to validate outputs from DESeq2 because it takes into consideration factors that may influence the overall outcome. The function ancombc was run on the phyloseq class object as input with parameters set as follow: p_adj_method = “fdr”, alpha = 5%, global = T and prv_cut = 0.50.

## 4 Results

### 4.1 Insecticide Resistance Profile of *Anopheles funestus* From Mibellon

**FIGURE 2:**
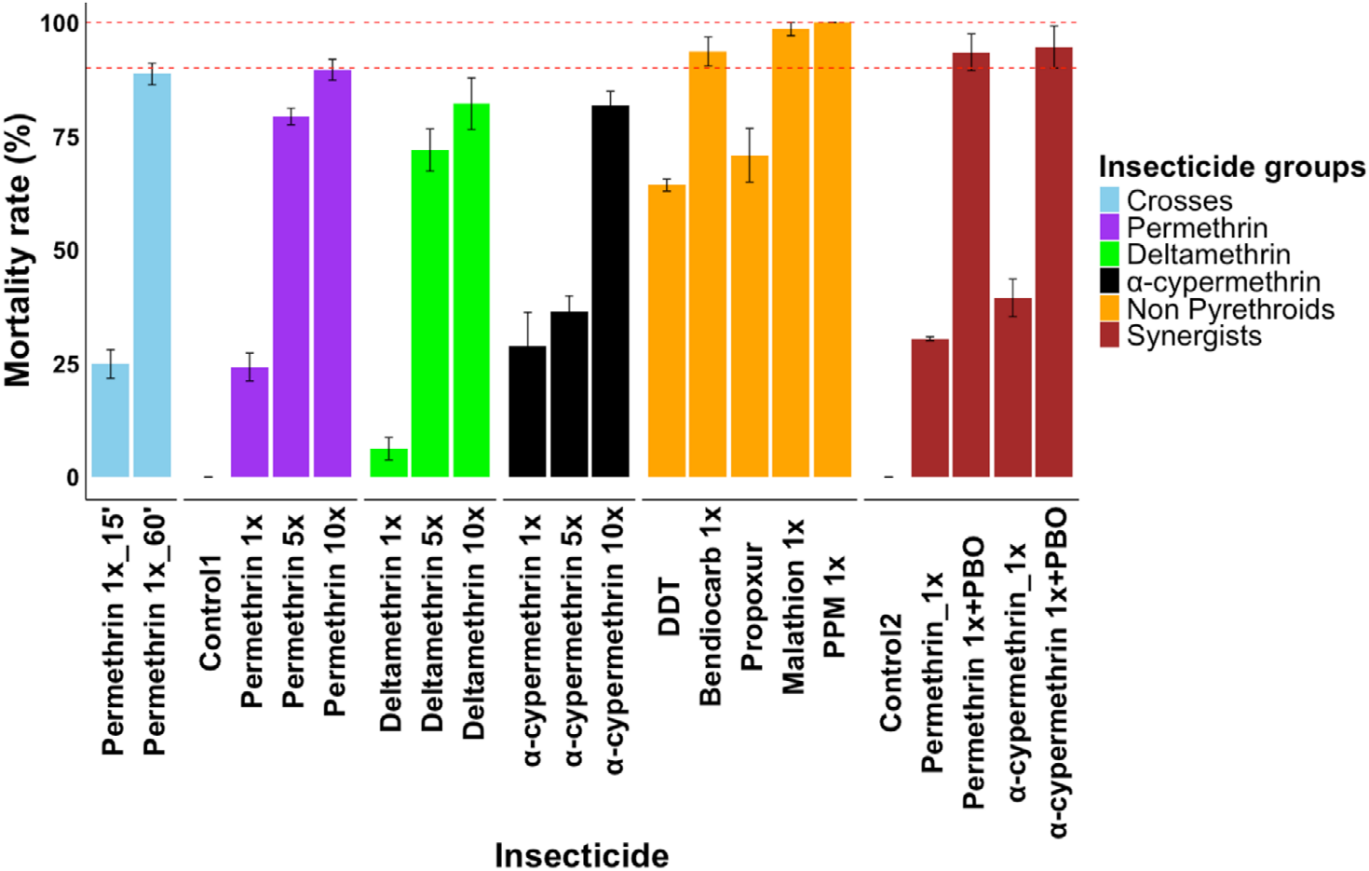
Susceptibility profile of *An. funestus* from Mibellon to the four classes of insecticides.

*An. funestus* population collected in Mibellon was resistant to all classes of insecticides except organophosphates Malathion 1× and Pirimiphos‐methyl 1× (mortality rate > 98% ± 1.44%). This population showed moderate to high resistance intensity (79.32% ± 1.81%, 71.94% ± 4.66%, 36.48% ± 3.35% to permethrin, deltamethrin and alpha cypermethrin 5×, respectively) and high resistance intensity to all pyrethroids type I and II (89.46%±2.29%, 82.13%±8.67%, 81.66% ± 3.20% for permethrin, deltamethrin and alpha cypermethrin 10×, respectively) indicating that they are super‐resistant. It was noticed that hybrid genetic crosses were still resistant to permethrin1× at generation F_2_, (88.67% ± 2.31%). Additionally, synergist assay with PBO significantly restored the susceptibility to pyrethroids insecticides indicating that the major resistance mechanism driving resistance escalation in this population is mediated by metabolic resistance mechanisms through cytochrome P450s genes (Figure 2).

### 4.2 Overview of Mapping and Coverage Statistics Across *Anopheles funestus* Phenotypes

PoolSeq produced paired‐end 150 × 2‐bp reads with varying coverage and mapping rates across samples. The read count ranges from 164 million for Dead 10× pool 1 to 273 million for Control unexposed pool 1, with an average of 188 million reads (Table S2). After trimming and filtering based on read pairing, sequence quality, and mapping quality, more than 60% of reads successfully mapped to the *An. funestus* FUMOZ reference genome. The majority of the reads aligned in pairs (> 90%), while around 5% for each pool were singletons. Sequencing covered over 98% of the genome for each sample, with varying mean depths among them. Mean depth ranged from 35× for Mibellon 2014 F_0_ population to 113× for Control unexposed pool 1, with an average of 82× (Table S3).

### 4.3 Principal Component Analysis

**FIGURE 3:**
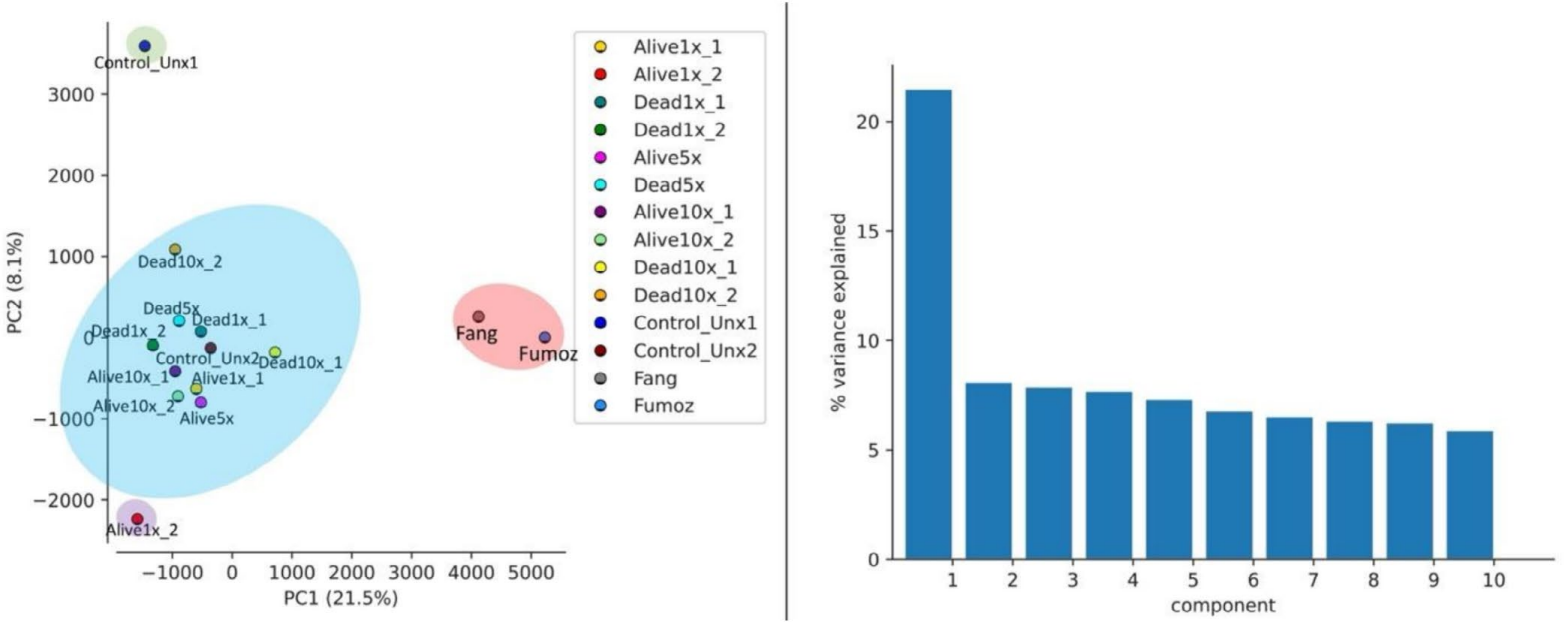
PCA plot of field F_1_ and genetic crosses *An. funestus*.

Principal component analysis (PCA) showed that the variance observed among samples was explained by PC1 (21.5%) (Figure 3 right). Examining the first two principal components (PC1 and PC2) of the field G1 Mibellon phenotypes, the samples formed two major clusters: the alive and dead Mibellon mosquitoes were genetically similar, whereas the laboratory strains (FANG and FUMOZ) clustered separately (Figure 3 left). This clustering was in broad agreement with their geographical locations separating Central Africa (Cameroon) from Southern Africa (Angola and Mozambique) mosquito populations. Looking at the Mibellon alive and dead 1×, 5× and 10× phenotypes as well as the control unexposed critically, we can notice a cryptic phenotype structure where all the phenotypes are overlapped and clustering together except the control unexposed 1 and alive 1×_2 who are outliers. This indicates that they still have similar genetic makeup despite the phenotyping. Stronger differentiation between those phenotypes versus laboratory strains was noted (Table S4). The similar observation was made for the genetic crosses where the hybrid crosses alive highly resistant and dead highly susceptible were very close to each other, whereas the laboratory strains were clustered in their own. Correlation analyses for both field samples and genetic crosses support the results of the PCA analysis as high pairwise *F*_*ST*_ values ranging from 0.25 to 0.32 were obtained when comparing any of the phenotypes to laboratory strains, indicating moderate genetic differentiation with potential isolation (Figures S3 and S4). Very low or no differentiation (*F*_*ST*_ ranging between 0.001 and 0.006) was observed between alive and dead phenotypes at various concentration level, not surprising as they are population from the same locality with similar genetic background, reared in similar conditions and environmental context despite exposure to insecticide (Tables S4 and S5).

### 4.4 Comparative Genomics Failed to Detect Genetic Loci Associated With Resistance Escalation in *Anopheles funestus* From Mibellon Using 1×, 5× and 10× Phenotypes

**FIGURE 4:**
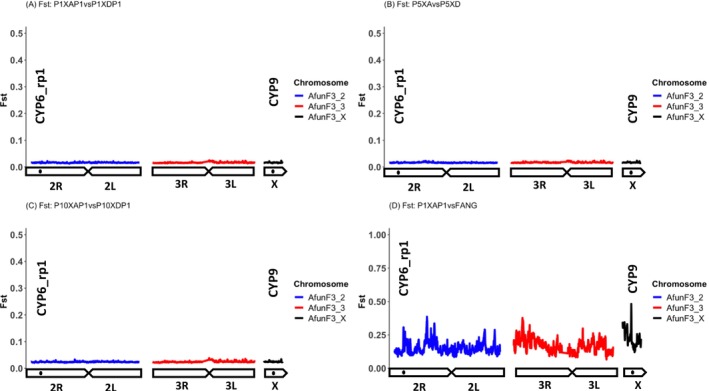
Pairwise *F*_*ST*_ genetic differentiation between alive and dead 1×, 5× and 10× *An. funestus* population from Mibellon.

To identify peaks of divergence potentially explaining the resistance escalation in F_1_ Mibellon mosquito population, pair‐wise *F*_*ST*_ (for the relevant comparisons including alive versus dead 1×, alive versus dead 5×, alive versus dead 10×, dead 1× versus alive 5× and dead 1× versus alive 10× for all the pools) was calculated for every SNP in popoolation2 and summarised in various overlapping windows sizes. Similar and consistent patterns were seen for most of the comparison with notable absence of selection signals genome‐wide (Figure 4A–C) highlighting the complexity of using field mosquito populations where the resistance is already high to detect signals of evolutionary selection. This pattern aligns with and validates the PCA findings, with consistency noted among the pools analysed.

In parallel, pairwise comparisons between alive 5×, 10× and dead pools revealed a stronger signal emerging at 6 Mb post the *rp1* locus on Chromosome 2. This signal manifested consistently in most comparisons between alive and dead pools at 10× concentrations, and in certain cases between dead 1× versus alive 5× and 10× alive (Figure S5). Intriguingly, this signal coincided with the α‐mannosidase gene (*AFUN009261*), not previously identified as a putative insecticide resistance gene.

However, pairwise comparison of all these phenotypes (alive and dead 1×, 5× and 10×) versus FANG yielded several genome‐wide perturbation of high background *F*_*ST*_ values with the strongest one coming up around the known rp1 QTL locus as well as the *CYP9* where is located *CYP9K1* (Figure 4D and Figure S6). Absence of strong signal of divergence in pairwise comparison among F_1_ field phenotypes support the high resistance pattern of this malaria vector population in line with the WHO bioassay findings. Such situation is preventing the detection of key loci/drivers of pyrethroid resistance evolution in Mibellon and poses a real challenge in deciphering the complex genomic evolution of pyrethroid resistance escalation drivers in this important malaria vector to inform governments and control programs on future actions to be taken to drive malaria towards elimination.

Facing this challenge, we used genetic crosses and time series PoolSeq data using alive highly resistant and dead highly susceptible from hybrid crosses and Mibellon 2014 versus 2021 F_1_ unexposed control population from Mibellon to confirm if the signals observed using F_1_ field phenotypes versus the laboratory strains are associated to resistance escalation or if other prominent signals could explain resistance evolution in *An. funestus*. Time series data allows for capturing temporal changes between two time points. For instance, in the 2014 population, no selection was documented in Mibellon (Weedall et al. 2020a), while VCTs and extensive pesticide use for agriculture were deployed there subsequently. So, we hypothesised that these stimuli may have selected certain resistant‐related loci in the Mibellon population over time, allowing them to now survive higher doses of pyrethroids (5× and 10×).

**FIGURE 5:**
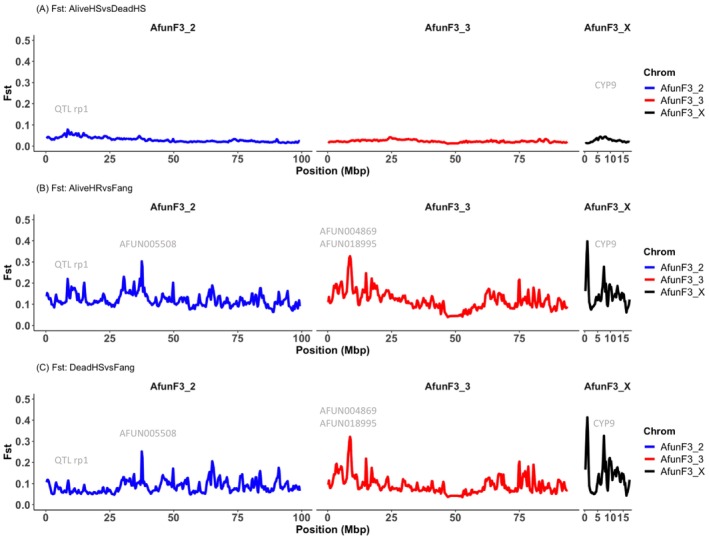
Pairwise *F*_*ST*_ genetic differentiation among *An. funestus* highly resistant and highly susceptible genetic crosses.

Weak pattern of genetic differentiation (*F*_*ST*_ < 0.1) was observed between the genomes of highly resistant and highly susceptible individuals, highlighting two major known resistance‐related loci around 8 Mbp on both chromosomes 2R and X, the *CYP6* and *CYP9* (Figure 5A). However, when comparing each of the crosses phenotypes to the fully susceptible FANG population, we observed the emergence of two prominent peaks with stronger mean *F*_*ST*_ values at the *rp1* and *CYP9* loci in the genomes of highly resistant individuals compared to FANG (Figure 5B), as opposed to highly susceptible individuals compared to FANG (Figure 5C). This indicates the significant contribution of both loci to the pyrethroid resistance escalation in the Mibellon population.

The α‐mannosidase peak detected in the F_1_ field samples was also found in genetic crosses but at lower *F*_*ST*_ value. This second approach using genetic crosses was successful in detecting two major resistance‐related loci although with low *F*_*ST*_ values overlapping with major pyrethroids resistance loci containing major cytochrome P450 genes. Additionally, temporal genomic analysis was done to strengthen the findings observed with genetic crosses and to capture the genomic changes in *An. funestus* populations over time.

**FIGURE 6:**
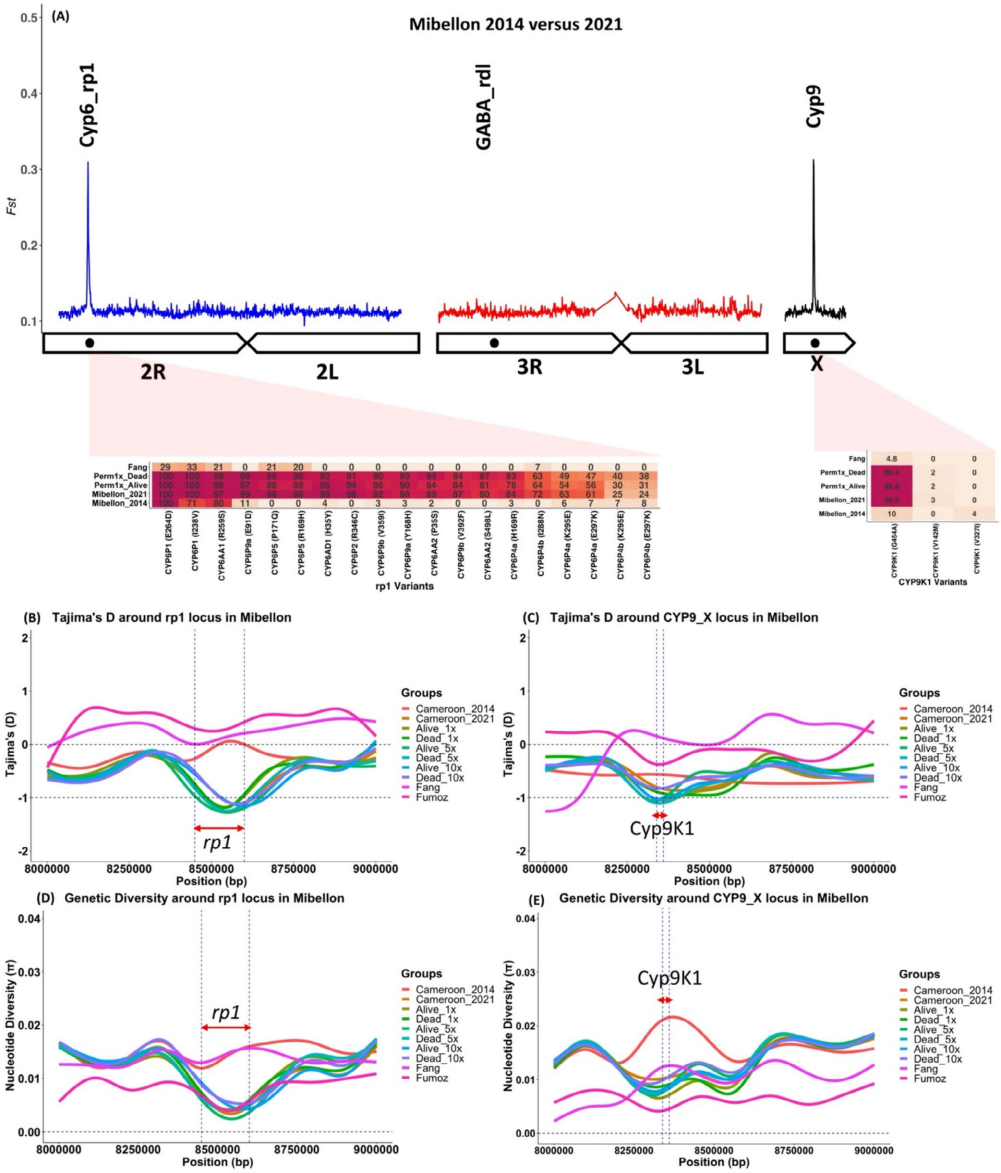
Selective sweeps spanning the *rp1* and *Cyp9 k1* gene in *An. funestus* in Mibellon. (A) Schematic pairwise F_ST_ genetic differentiation among field F_0_ An. funestus collected between 2014 and 2021 in Mibellon. (B–E) Genetic diversity and Tajima's D indices indicating selective sweeps spanning the rp1 and CYP9K1 loci in An. funestus from Mibellon. Both estimates were computed in overlapping windows of 1 kb moving in steps of 0.5 kb after subsampling to uniform the coverage to 20×. The horizontal red arrow in between the blue vertical lines indicates the start and the end of rp1 locus in B and D while the arrow spanning the CYP9K1 gene in C and E indicates the start and end of the CYP9K1 gene.

Temporal genomic comparison of *An. funestus* population collected in Mibellon between 2014 and control unexposed 2021 clearly identified for the first time in Mibellon, Cameroon two major genomic regions with high background *F*_*ST*_ values potentially driving resistance escalation in this important malaria vector in Mibellon. It was noticed the emergence of two well‐known resistance‐associated loci on chromosome 2R (*rp1* QTL) and X chromosome (*CYP9* locus) (Figure 6A). These findings validated and align with those from the genetic crosses and F_1_ field phenotypes versus FANG indicating that pyrethroid resistance escalation in *An. funestus* population in Mibellon is potentially controlled by the temporal co‐emergence of two P450s‐based resistance‐related loci. Within the *rp1* locus, the emergence and fixation of P450‐based single nucleotide polymorphisms (SNPs) in this population were responsible for the observed signal. These SNPs were found at higher frequencies or were fixed in both alive and dead phenotypes of highly resistant individuals, regardless of the concentrations, compared to highly susceptible individuals in the 2021 population. In contrast, these SNPs remained absent or at very low frequencies in the FANG fully susceptible population and in the population from 2014 indicating their important role in super‐resistance (Figure 6A and Figure S9).

A similar pattern was observed within the *CYP9* locus, where the temporal emergence and fixation of a single amino acid change (G454A) in the only P450 gene of this region (*CYP9K1*) induced the observed signal on this sex‐linked chromosome. The allele frequency of the mutant allele (A454A) was fixed in both alive and dead phenotypes (1×, 5× and 10× concentrations) and in the 2021 control unexposed population. It was also higher in highly resistant individuals compared to highly susceptible genetic crosses, while remaining at very low frequencies in the FANG and 2014 populations.

Further validation of these resistance‐related loci through comparisons between the Mibellon populations of 2014 and control unexposed 2021 versus the FANG population confirmed the emergence of the two major loci in Mibellon 2021. This contrasted with the findings in Mibellon 2014 versus FANG, where the signals were either absent or weaker. These results underscore the significant role of both loci in insecticide resistance evolution in the *An. funestus* population in Mibellon.

### 4.5 Population Genomic Analyses Detected Signatures of Selective Sweeps Associated With the Identified Genetic Loci Potentially Explaining Resistance Escalation in *Anopheles funestus* in Mibellon

Evidence of positive selection was detected through computation of genome‐wide Tajima's D in non‐overlapping windows of 50 kb, moving in steps of 25 kb. Further fine‐scale resolution was achieved by zooming in around both resistant‐related loci using non‐overlapping windows of 1000 bp across all phenotypes and populations. Genome‐wide median Tajima's D values were negative across all the chromosomes for all the populations/phenotypes except in dead highly susceptible and Mibellon 2014 samples where it was higher (Figure S5). Furthermore, FANG and FUMOZ laboratory strains show higher level of Tajima D (D > 0) suggesting no evidence of selection as there are laboratory strains not suggested to selective pressure. Lower values of Tajima's D were seen across sex‐link X chromosome for all the field and hybrid population compared to autosomes potentially indicating strong selection happened on this chromosome compared to others. Mibellon 2014 population showed higher median Tajima's D compared to Mibellon 2021 and field F_1_ populations indicating no or less sign of selection in 2014 *Anopheles funestus* population, with positive selection in 2021 population (unexposed) from this locality (Figure S5).

Zooming in around the *rp1* cluster for good resolution in windows of 1 kb revealed consistently negative median Tajima's D values observed across the entire *CYP6* locus in all field F_1_ phenotypes, and the Mibellon 2021 population, but not in the 2014 Mibellon, FANG and FUMOZ populations. Interestingly, Tajima's D values were consistently negative specifically within the rp1 region (as shown in Figure 6B between the blue dashed vertical lines), indicating a pronounced valley not only in reduced Tajima's D values but also in reduced genetic diversity (Figure 6D) across all field phenotypes and the 2021 Mibellon population, but not in laboratory strains or the 2014 Mibellon population. These findings suggest evidence of a selective sweep specifically affecting this locus, with the Mibellon 2014 population closer to equilibrium (suggesting balancing selection), and the FANG and FUMOZ populations remaining in equilibrium. A similar pattern was observed around the *CYP9K1* gene, where evidence of positive selection was induced by strongly negative Tajima's D values across all samples compared to the 2014, FANG, and FUMOZ samples (Figure 6C). This pattern was accompanied by reduced genetic diversity between the 2014 and 2021 *Anopheles funestus* populations from Mibellon (Figure 6E).

Negative Tajima's D in both alive and dead phenotypes could potentially suggest that this *Anopheles funestus* population has recently experienced a selective sweep that reduced the genetic diversity and this poses a great challenge in detecting specific variant driving resistance evolution using field F_1_ sample as the variant of interest may have been selected to fixation in both phenotypes.

### 4.6 Non Synonymous Polymorphisms Associated With Pyrethroid Resistance Escalation in *Anopheles funestus*

Non‐synonymous polymorphisms, also known as non‐synonymous single nucleotide polymorphisms (ns‐SNPs) are genetic variations that result in a change in the amino acid sequence of the encoded protein and has been shown to drive insecticide resistance in malaria vectors including *Anopheles funestus* in Benin and Cameroon (Tchouakui et al. 2019; Riveron et al. 2014). SNP calling detected significant and novel polymorphisms around the resistance‐related loci. In all, 127,742 SNPs were detected among which 838 ns‐SNPs spread over *CYP6*‐*CYP9* loci. After applying comprehensive allelic frequencies, *p*‐values‐based filters and considering those located within the active site or the substrate biding pocket of the corresponding genes, about 35 ns‐SNPs were found to be meaningful (Figure S8). Among those ns‐SNPs, P450s‐based genes were the top harbouring significant ns‐SNPs including *CYP9K1* on the X chromosome, *CYP6AD1*, *CYP6P2*, *CYP6AA2*, *CYP6P9a, CYP6P9b*, *CYP6P5*, *CYP6P4a* and *CYP6P4b* on the autosome 2R, all of them being located within the rp1 region known to explained 87% of the resistance in *Anopheles funestus*. The strongest and most consistent ns‐SNPs associated to super‐resistance was the *CYP9K1* (G454A, freq = 96.48% and *p* = 5.56E‐139), followed by novel SNPs on *CYP6AD1* (H35Y, freq = 97.35% and *p* = 2.74E‐48), *CYP6P2* (R346C, freq = 97.74% and *p* = 3.09E‐46), *CYP6AA2* (P35S, freq = 89.23% and *p* = 1.23E‐43), *CYP6P9a* (E91D, freq = 98.69% and *p* = 4.07E‐42), *CYP6P5* (V14L, freq = 97.49% and *p* = 7.32E‐42) and *CYP6P9b* (V392F and V359I, freq > 80% and *P* ~ 4.20E‐41). All these SNPs were significantly found at higher frequencies or fixed in alive and dead field F1 phenotypes (1×, 5× and 10×), at higher frequencies in alive highly resistant compared to dead highly susceptible crosses. Temporal allelic frequency analyses confirmed that all these SNPs have emerged over time as they were either absent or at very low frequencies and not significant (Allele frequency < 12%, *p*‐value < 0.056) in 2014 mosquito population compared to the 2021 population (Figure S9). *CYP9K1* (G454A) with other SNPs emerged within 7 years to become fixed in the population as it was at very low frequency and non‐significant (freq = 10% and *p* = 0.12) in 2014 compared to 2021 population (freq = 95% and *p* = 5.44E‐14) (Figures S8 and S9). SNPs from the rp1 locus were not significant and completely absent in the fully susceptible FANG strain (freq = 0%, *p* value = 1) confirming their potential role in resistance escalation in Mibellon. The frequencies of all these variants were increasing temporally indicating that increase allele frequency had potentially driving super‐resistance in field *An. funestus* population. Other novel and consistently significant SNPs found at moderate to high frequencies include *CYP6P4a* and *CYP6P4b* (H169R, I288N, K295E, and E297K), Carboxylesterases (AFUN015793 (R480S, S307Y) and AFUN15787 (A445T, Q443L)) and *GSTe2* (L119F and K146T) (Figures S8 and S9; Table S9).

### 4.7 Signatures of Complex Genomic Evolution Associated With Both *rp1* and *CYP9* Loci

Computational‐based large structural variant analyses and visualisation of alignment files in IGV reveal distinct pattern indicative of a transposon insertion on chromosome 2R, located downstream of *CYP6P9b* and upstream of *CYP6P5*. This transposon spans 4.3 kb, possesses an insertion sequence of ‘CCAAATGTACA’ and precisely located at positions 8556411–8556420 (Table 1 and Figure S11A,B). The transposable element (TE) has been definitively identified across a spectrum of *An*. *funestus* phenotypes, demonstrating robust consistency in both alive and dead mosquitoes. Temporal analysis indicates the absence of this transposable element (TE) in the 2014 population, while it has emerged in the 2021 population, suggesting its possible implication in resistance evolution in *An. funestus* in Mibellon. In‐depth analyses with INSurVeyor support the TE's consistency in 2021 population, revealing, for instance, 114 discordant reads (92 forward, 22 reverse), 43 supporting reads (26 forward, 17 reverse), and substantial coverage of 387 at the transposon site (124 forward, 155 reverse).

**TABLE 1:** Structural variants polymorphism in the *CYP6* and *CYP9* genomic regions.

Population	Year	Size	Position	SVs_type	Description
Mibellon	2021	40	rp1:8560667–8563170	Duplication	Tandem duplication (2.5 kb) spanning partial *CYP6P4a* and partial *CYP6P4b*
Mibellon	2021	40	rp1: 8536335–8539804	Duplication	Tandem duplication (3.4 kb) spanning *AFUN015797* and *AFUN015793*
Mibellon	2021	40	rp1:8528585–8535070	Duplication	Tandem duplication (6.4 kb) spanning *CYP6AA1* and partial *CYP6AA2*
Mibellon	2021	40	rp1:8528384–8545317	Duplication	Tandem duplication (16.8 kb) spanning the entire *CYP6AA1*, *CYP6AA2*, 2× CEs, *AFUN008357* and partial *CYP6P9a*
Mibellon	2021	40	rp1:8546959–8553509	Deletion	Deletion (6.5 kb) between *CYP6P9a* and *CYP6P9b*
Mibellon	2021	40	rp1: 8556411–8556420	Mobile element insertion	Transposon insertion of 4.3 kb between *CYP6P9b* and *CYP6P5*
Mibellon	2021	40	CYP9K1: 8338433–8338440	Mobile element insertion	Huge transposon insertion upstream *CYP9K1* gene with incomplete assembly sequence length
Mibellon	2021	40	CYP9: 8337671–8343486	Duplication	Potential tandem duplication (5.8 kb) of *CYP9K1* gene

In addition, we reported a large transposon insertion similar to that identified above within the *CYP9* locus upstream the *CYP9K1* gene. The program used to assemble the entire sequence of this transposable element (TE) allowed us to uncover just 849 bp with a pattern indicative of a large TE insertion. This novel TE, inserted upstream of the *CYP9K1* gene at position 8338432, is characterised by an insertion sequence of “CAAATTTC” (Table 1 and Figure S11C,D). It was absent in the reference genome, as well as in the 2014 and FANG populations, but consistently present in all the field samples and the 2021 population, suggesting its potential implication in resistance evolution in the Mibellon population. INSurVeyor analysis supports the consistency of the TE with substantial number of discordant reads, supporting reads, and high coverage depth at the transposon site in G1 and 2021 population. Taken together, these findings underscore the stability and integration of the transposon within the *An*. *funestus* genome.

Besides the TE, our study unravels compelling evidence of diverse duplications and large insertions/deletions (indels) dispersed across both the rp1 and *CYP9* loci, potentially associated with the evolutionary plasticity of *An*. *funestus* in Mibellon. Notably, certain duplications and indels were previously identified in 2014 population (Weedall et al. 2020a), such as a 6.4 kb duplication spanning the entire *CYP6AA1* and partial *CYP6AA2* (Figure S11E), a 16.8 kb duplication spanning the entire *CYP6AA1*, *CYP6AA2*, two carboxylesterases (CEs), *AFUN008357*, and partial *CYP6P9a* (Figure S11F), as well as a 6.5 kb fixed intergenic deletion between *CYP6P9a* and *CYP6P9b* (an insertion in southern African populations) (Figure S11E,H) (Weedall et al. 2020a). Using tool‐based approach with Smoove, this current study unveils three novel duplications not previously reported within the rp1 region. The first one is a notable 2.5 kb duplication between position 8560667–8563170, partially spanning *CYP6P4a* and *CYP6P4b* paralogs (Figure S11I), previously identified as overexpressed in Ghana population, was identified in all field samples, including those collected in 2021. This duplication, impacting both strands, was supported by at least 12 reads and present on 12 paired‐end reads with high coverage, showcasing its robustness. The second one is a 3.4 kb duplication between position 8536335–8539804 spanning the two carboxylesterases (*AFUN015797* and *AFUN015793*) garnered support from both strands (Figure S11J), with 39 supporting and paired‐end reads. The third duplication is a 3.4 kb between position 8537818–8541289, spanning partial carboxylesterase (*AFUN015793*), while supported by only four reads. Interestingly, these structural variations were observed at or near fixation in all field G1 population and 2021 populations, but were absent in FANG, FUMOZ, and 2014 populations, or present on very low frequencies. This suggests a potential adaptive response to selection pressure by the *An*. *funestus* population in Mibellon.

Further inspection of the *CYP9K1* gene within the *CYP9* cluster suggests a potential gene tandem duplication of 5.8 kb (Table 1 and Figure S11), adding another layer of complexity to the resistance mechanism besides the SNPs and the large TE insertion identified upstream.

### 4.8 Evidence of Copy Number Variations (CNVs) Confirms the Duplication of *CYP9K1* in the *Anopheles funestus* Population From Mibellon

**FIGURE 7:**
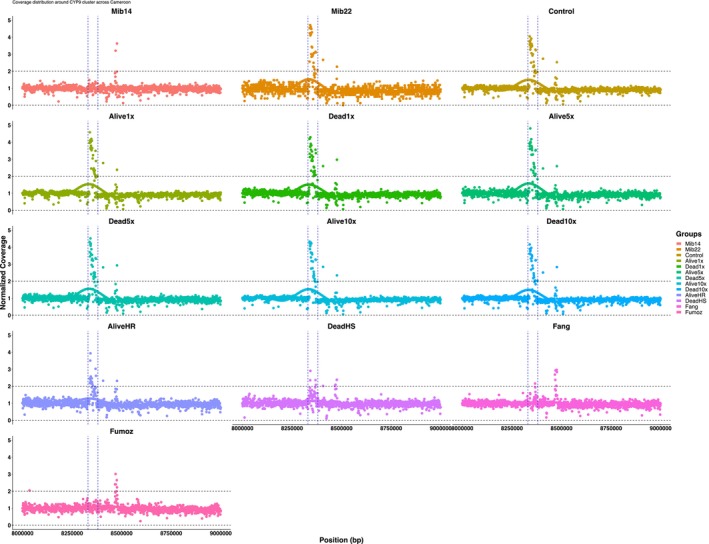
Evidence of CNVs spanning the *CYP9* locus around *CYP9K1* in *An. funestus* population from Mibellon, Cameroon in relation to resistance intensity. Each dot on the plot represents a window of 1000 bp.

To confirm the presence of the 5.8 kb tandem duplication spanning the entire *CYP9K1* gene, coverage analysis was conducted across all field F_1_ phenotypes, genetic crosses, and populations from 2014, 2021, FANG, and FUMOZ (Figure 7). Coverage depth at each locus was computed, normalised per GC content and median coverage depth, and summarised in non‐overlapping windows of 1000 bp. Visualisation per phenotype was performed using ggplot2. Results indicate the absence of copy number variations (CNVs) in the 2014, FANG, FUMOZ, and ostensibly highly susceptible populations. However, evidence of at least one additional copy of the *CYP9K1* duplication allele was consistently found in both alive and dead phenotypes, as well as in the 2021 unexposed population (seen between the two vertical blue dashed lines on Figure 7) suggesting the emergence and fixation of CNVs over time.

### 4.9 Polymorphism Analysis of *CYP6P9b* and Combined Genotyping of 4.3 Kb and *CYP9K1* (G454A) bi‐Markers in Highly‐Resistant *Anopheles funestus* in Mibellon

Particular attention was given to *CYP6P9b* gene which result from a fixed duplication of *CYP6P9* in *Anopheles funestus* compared to other malaria vector species (Wondji et al. 2009). Polymorphisms analysis using Sanger sequencing of this gene were comparatively assessed between two hybrid phenotypes by focusing primarily on validating the nonsynonymous SNPs found in PoolSeq data. We found two substantial SNPs associated to the highly resistant phenotype within the *CYP6P9b* gene (V392F and V359I). The allelic frequencies of these SNPs were higher in the highly resistant (HR) and lower in the highly susceptible (HS) crosses (Figure S10A,B,E), confirming results from the PoolSeq analysis. Polymorphism analysis indicated that the *CYP6P9b* gene is diverse among crosses, with a similar number of haplotypes (9 for HR and 10 for HS) and comparable haplotype diversity (0.88 for HR and 0.92 for HS). Reduced nucleotide diversity and a non‐significant Tajima's D were observed in the HR compared to the HS crosses, which could potentially indicate signs of selection, with evidence of rare alleles in both phenotypes (Table 2). The haplotype network revealed a unique dominant haplotype shared between HR and HS, with a predominance of the resistant phenotype (Figure S10D). The phylogenetic tree showed a highly diverse phenotype pattern, with overlapping phenotypes, although a cluster predominantly formed by the HR phenotype is noted (Figure S10C). A Fisher exact test was used to assess the association between the mutant allele of *CYP6P9b* (392F) and the phenotype using 10 alive and 10 dead *An. funestus* from genetic crosses. Results indicated a significant association between the mutant allele (T/T) and the highly resistant phenotype (Figure S10F), with homozygote resistant mosquitoes having a fourfold higher chance of surviving permethrin exposure (1×) than homozygote susceptible mosquitoes (OR = 4, *p* = 0.033). This suggests that the *CYP6P9b* (392F) allele is associated with a highly resistant phenotype in the Mibellon locality.

**TABLE 2:** Genotyping of 4.3 kb SV and *CYP9K1* molecular markers in *An. funestus* genetic crosses from Mibellon.

Genotypes	OR [95% CI]	*p*
*4.3 kb SV*		
SV + SV+ vs. SV − SV−	78.5 [8.9–3855.6]	**9.599e‐08**
SV + SV+ vs. SV + SV−	3.9 [0.44–190.7]	0.2501
SV + SV− vs. SV − SV−	21.4 [5.9–95.5]	**2.069e‐08**
*CYP9K1*		
RR vs. SS	43.9 [4.3–2347.8]	**6.411e‐05**
RR vs. RS	4.4 [0.5–210.4]	0.2473
RS vs. SS	11.2 [3.2–51.1]	**8.49 2e‐06**
*GSTe2_L119F*		
RR vs. SS	6.4 [0.6–340.4]	0.1458
RR vs. RS	2.8 [0.2–149.5]	0.6351
RS vs. SS	2.3 [0.9–6.1]	0.0795
*CYP9K1/SV*		
RR/SV + SV+ vs. SS/SV − SV−	13.1 [0.1–1460.3]	0.1857
RR/SV + SV+ vs. RS/SV + SV−	0.1 [0.0013–12.4]	0.2490
RS/SV + SV− vs. SS/SV − SV−	**126.6 [11.7–6981.4]**	**5.82e‐08**
*GSTe2_L119F/SV*		
RR/SV + SV+ vs. SS/SV − SV−	14.5 [0.1–1611]	0.1700
RR/SV + SV+ vs. RS/SV + SV−	0.25 [0.003–24.1]	0.4052
RS/SV + SV− vs. SS/SV−SV−	69.6 [6.9–3737.4]	**1.945e‐06**
*9 k1/GSTe2/4.3 kb*		
RR/RR/SV + SV+ vs. SS/SS/SV − SV−	NA	NA
RS/RS/RS vs. SS/SS/SV − SV−	45.8 [2.8–3401.8]	**0.0011**
RR/RR/RR vs. RS/RS/SV + SV−	NA	NA
*9 k1 and GSTe2*		
RR/RR vs. SS/SS	NA	NA
RR/RR vs. RS/RS	NA	NA
RS/RS vs. SS/SS	30.7 [3.4–1545.8]	**0.0001**

*Note:* RR, RS and SS denote the homozygous resistant, heterozygous and homozygous susceptible genotypes, respectively, for *CYP9K1* (G454A) and *GSTe2* (L119F). SV+/SV+, SV−/SV− and SV+/SV− represent the homozygous resistant, heterozygous and homozygous susceptible genotypes, respectively, for the 4.3 kb structural variant. OR [95% CI] refers to the odds ratio with its Corresponding 95% credible interval. The values shown in bold in Table 2 indicate statistically significant comparisons (p < 0.05).

Alignment and visualisation in IGV of field F_1_ *Anopheles funestus* population to the 4.3 kb SV sequence showed that this variant was fixed in all the field samples with high coverage depth, even in F_0_ Mibellon 2021 population but absent in 2014, FANG and FUMOZ. Additionally, the *CYP9K1* (G454A) from the *CYP9* cluster was of interest because of its increased allelic frequency of the mutant to fixation between 2014 and 2021 in *An*. *funestus* in Mibellon. Because the two variants were already fixed in the population, we took advantage of the genetic crosses generated as well as the DNA‐based tools previously designed to assess the combined impact of the two variants located in two different major genomic regions on super‐resistance in *An*. *funestus* in Mibellon Djoko Tagne et al. 2024a. We additionally genotype *GSTe2* (L119F) marker following previous studies (Riveron et al. 2014). Genotyping of 4.3 kb SV in 88 *Anopheles funestus* hybrids (44 highly resistant and 44 highly susceptible) post exposure to pyrethroids indicated a strong association between the 4.3 kb SV and highly resistant phenotype with mosquitos being homozygote (SV+/SV+; OR = 78.5, CI [8.9–3855.6], *p* = 5.9e‐08) and heterozygote (SV+/SV‐; OR = 21.4, CI [5.9–95.5]; *p* = 2.069e‐08) for the structural variant significantly surviving post exposure to pyrethroids than homozygote susceptible (SV‐/SV‐). Same results were found with *CYP9K1* mutant allele strongly associated with highly resistant phenotype with homozygote and heterozygote for the mutant variant being more able to survive than homozygote bearing the wild type allele (Table 2). No association was found for *GSTe2* using genetic crosses. The combination of the two markers increases ability of mosquitoes to survive exposure to pyrethroids than just having one of the two markers (Table 2). Indeed, possessing a single copy of each resistant marker (SV+/SV‐/RS+/RS‐) is sufficient for a mosquito to become highly resistant, compared to just having one or two copies of individual markers (OR: 126.6; CI [11.7–6981.4], *p* = 5.82e‐08).

### 4.10 Overview of Microbial Composition Among Phenotypes and Treatments

Kraken2‐based microbial classification showed that over 9% of reads per sample aligned to the database, with classified reads ranging from 9.56% to 64.35%. Bacterial reads were the most abundant, peaking at 63.92% in control unexposed and lowest at 7.12% in permethrin 5× dead. Viral reads were minimal (0.003%–0.009%), while no fungal, protozoan, or artificial sequences were detected, indicating no contamination (Table S7). Following comprehensive filtering and the removal of contaminants from all our samples, our analysis revealed a striking dominance of the Bacteria Kingdom prevailing at a remarkable 99.34% abundance (Table S8). This kingdom encompasses a diverse array of microorganisms, consisting of 13 Phyla, more than 20 classes, orders, more than 135 families, and genera, and a wealth of species.

Among the discernible Phyla, *Proteobacteria*, *Actinobacteria*, *Firmicutes*, and *Bacteroidetes* emerged as the most abundant, collectively representing a substantial 98.42% of the total microbial abundance (Table S8). Notably, *Proteobacteria* assumed a position of paramount importance, constituting an impressive 56.12% of the entire microbial landscape. *Actinobacteria* followed suit at 33.46%, while *Firmicutes* and *Bacteroidetes* contributed 5.59% and 3.25%, respectively. These dominant phyla offer a glimpse into the prevailing taxonomic diversity within our samples.

At the class level, *Alphaproteobacteria*, *Betaproteobacteria*, *Actinobacteria* and *Gammaproteobacteria* emerged as the principal representatives, collectively accounting for over 88.97% of the total microbial abundance. These classes, within the *Proteobacteria* and *Actinobacteria* Phyla, play a pivotal role in shaping the microbial composition.

Our exploration of the order level uncovered *Burkholderiales*, *Micrococcales, Enterobacterales*, *Hyphomicrobiales*, and *Pseudomonadales* as the most influential and abundant orders, collectively constituting about 50% of the overall microbial population (Table S8).

**FIGURE 8:**
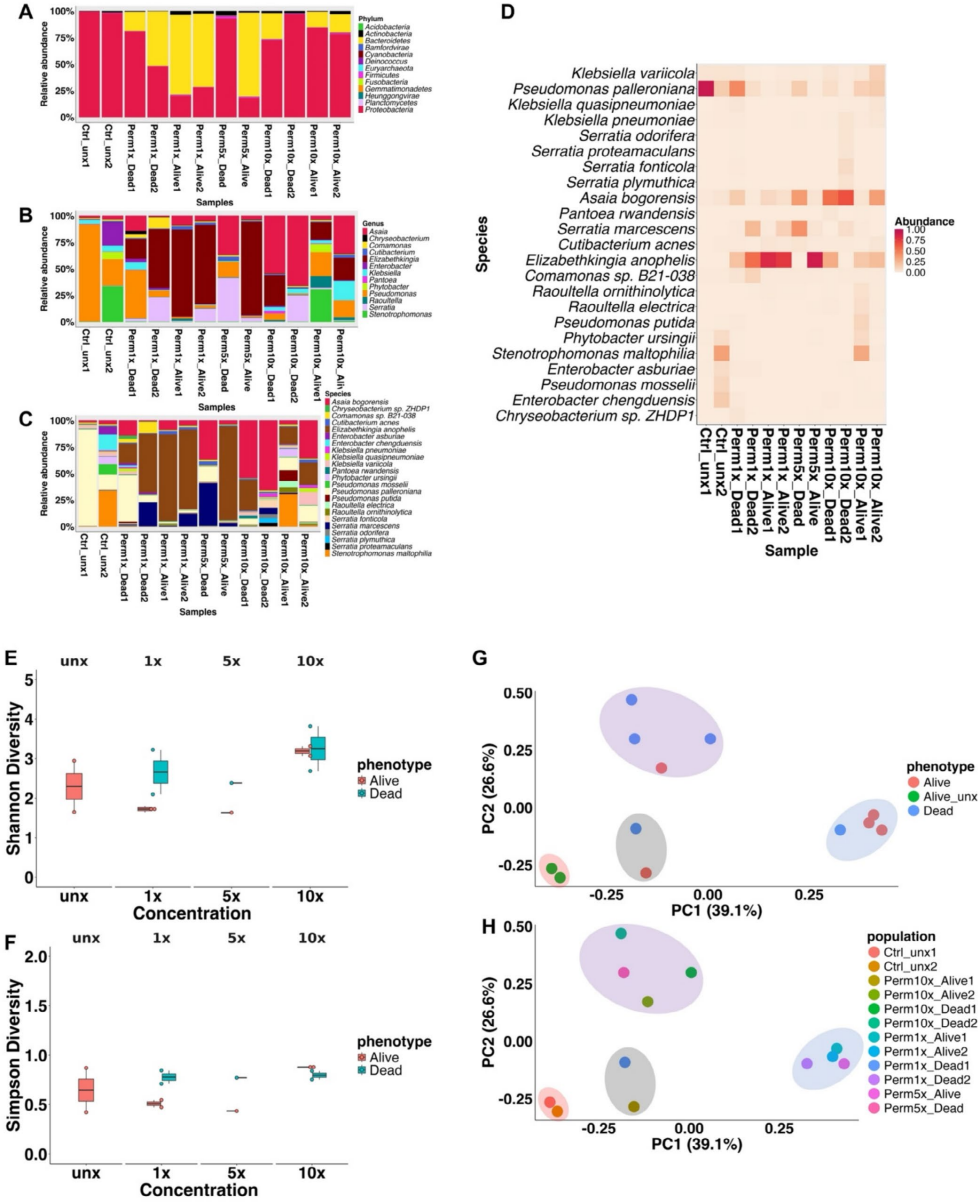
Microbial signature analyses in *An. funestus* from Mibellon. (A–C) represent relative abundance in relation to phenotypes/concentrations at phylum, genus and species levels; (D) represents the heatmap the top 2% abundant species according to various treatments; (E and F) represents alpha diversity (Shannon and Simpson) within treatments and (G and H) are between treatments beta‐diversity based on bray curti's dissimilarities.

Bacteria species were varying according to various treatments and phenotypes with *Elizabethkingia anophelis* mainly dominant in resistant phenotypes (Figure 8A–D) while *Asaia bongorensis* and species of the *Serratia* genus were dominant in susceptible phenotype (Figure 8A–D).

### 4.11 Microbial Community Diversity Analysis

Two alpha diversity statistics were performed to evaluate the diversity within sample. Alpha diversity Shannon index (diversity within sample) was higher in all the dead phenotypes compared to the alive phenotypes except for permethrin 10× samples where it was similar (Figure 8E,F). The Simpson diversity index supports the result obtained with the Shannon index. Although the indexes were higher in dead mosquito phenotypes, pairwise comparisons using Wilcoxon rank sum exact test of Shannon alpha diversity index for all possible scenario necessary for detection of microbial signature showed no significant difference among samples (phenotypes and concentrations) (FDR *p* > 0.05) suggesting similar microbial diversity among all the phenotypes and concentrations (1×, 5× and 10×) (Table 3).

**TABLE 3:** Wilcoxon test of Shannon diversity according to various phenotypes and concentrations.

Comparison	W	*p*	CI
Alive_1× vs. Dead_1×	0	0.5	−1.58; −0.29
Alive_5× vs. Dead_5×	0	1	−0.75; −0.75
Alive_10× vs. Dead_10×	1	1	−0.51; 0.38
Alive_1× vs. Control_unx	3	0.5	0.0075; 1.14
Alive_10× vs. Control_unx	0	0.5	−1.42; −0.368
Alive_10× vs. Dead_1×	2	1	−0.15; 1.21

Beta‐diversity (diversity between samples) was computed using Bray–Curtis dissimilarity distance test and principal coordinate analysis (PCoA) was done and displayed (Figure 8G,H). Microbiota of exposed mosquitoes were slightly different from those of unexposed control. This was evidenced by the fact Control unexposed (Ctrl_unx1 and Ctrl_unx2) are clustering away from exposed phenotypes. Bray–Curtis dissimilarity distance shows high overlap between exposed phenotypes and even between different treatments. Pairwise PERMANOVA test showed no significant differences in microbiota composition between each relevant comparison (Table 4). Pairwise comparisons between different sample concentration levels revealed no significant differences in microbiota composition, as exemplified by the comparison between permethrin 10× and permethrin 5× treatments (*F*‐value: 0.47; *R* ^2^: 0.19; *p*‐value: 1; *p* FDR: 1).

**TABLE 4:** Pairwise PERMANOVA beta diversity test among treatments.

Comparison	*F*	*R* ^2^	*p*	FDR
Control_unx vs. 1×	2.3203	0.37	0.067	0.4
Control_unx vs. 5×	1.1224	0.36	0.67	1
Control_unx vs. 10×	1.3632	0.40	0.33	1
1× vs. 5×	0.11413	0.028	1	1
1× vs. 10×	0.79868	0.168	0.53	1
5× vs. 10×	0.47227	0.198	1	1
Alive 1× vs. Dead 1×	1.58	0.44	0.33	1
Alive 1× vs. Dead 10×	2.12	0.51	0.33	1
Alive 1× vs. Dead 5×	1	1	1	1
Alive 10× vs. Dead 1×	0.95	0.32	0.66	0.4
Alive 10× vs. Dead 5×	1.38	0.58	0.66	0.4

### 4.12 Differential Abundance Analysis Unveils Microbial Associations With Pyrethroid Resistance Escalation

We conducted a comprehensive differential abundance analysis using DESeq2 to unveil the relationships between specific microbial species and the pyrethroid resistance in *Anopheles funestus* from Mibellon. Three distinct approaches were used to identify bacteria species associated with the traits of interest. Firstly, we contrasted all the alive (1×, 5× and 10×) phenotypes to the control unexposed samples to capture bacteria specifically or commonly abundant to the exposed (i.e., 1×, 5× and 10×) versus the unexposed and vice versa. Secondly, we compared the alive (1×, 5× and 10×) phenotypes versus the dead 1× and the alive 10× versus dead 5× and thirdly, we did pairwise comparisons among the exposed alive (1×, 5× and 10×) samples.

Our initial comparisons revealed that species within the *Elizabethkingia* genus were significantly more abundant in the alive phenotypes (1×, 5× and 10×) compared to the unexposed controls (Unx). The log2FC ranged from 5.70 for *Elizabethkingia ursingii* (alive 10× vs. Unx) to 10.58 for *Elizabethkingia meningoseptica* (alive 5× vs. Unx), with FDR values ranging from 2.64E‐05 to 7.60E‐21, respectively (Table S10). Interestingly, *Elizabethkingia anophelis* was significantly associated with resistant phenotypes at 1×, 5× and 10× with consistent read counts (Table S10).

Similarly, species from the *Cedecea* genus were significantly more abundant in alive (1×, 5×) phenotypes compared to controls. The log2FC varied from 3.85 for *Cedecea neteri* (alive 5× vs. Unx) to 4.79 (alive 1× vs. Unx), with FDR values ranging from 1E‐02 to 6E‐06.

Conversely, 13 bacterial species from the *Enterobacter* and *Comamonas* genera were significantly more abundant in unexposed samples compared to alive phenotypes. Absolute log2FC ranged from 2.04 for *Enterobacter ludwigii* (alive 10× vs. Unx) to 7.34 for *Enterobacter roggenkampii* (alive 1× vs. Unx), with FDR values between 4.E‐02 and 7.E‐08 (Table S10).

The second approach revealed several bacterial species that were significantly more abundant in the alive 1×, 5× and 10× phenotypes compared to the dead 1× phenotype. Notably, *Chryseobacterium* sp. PCH239 was more abundant in alive versus dead 1×, while species within the *Glutamicibacter* genus, *Brachybacterium* sp. SGAir0954, and *Weissella cibaria* were particularly abundant in alive 5× compared to dead 1× (Table S10). Furthermore, *Raoultella electrica*, *Stenotrophomonas maltophilia* , *Phytobacter ursingii*, and most *Pseudomonas* species were significantly associated with the alive 10× phenotype.

Conversely, most bacterial species within the *Serratia*, *Delftia*, some *Pseudomonas*, *Yersinia*, and *Xenorhabdus* genera were consistently more abundant in the dead 1× phenotype compared to alive 1×, 5×, and 10× (FDR < 0.05).

The third approach identified 11 bacterial species, with *Elizabethkingia anophelis* standing out as particularly noteworthy. This species was significantly more abundant in the 5× phenotypes compared to 1×, despite being more abundant in the 1× phenotypes compared to the 10× alive phenotypes (Table S10).

To strengthen the robustness of our findings, we employed analysis of the composition of Microbiome with bias correction 2 (ANCOM‐bc2), a powerful tool for differential abundance analysis which take into account features that may impact the overall outcome. This additional analysis confirmed most of the results obtained through DESeq2, fortifying our understanding of microbial associations with pyrethroid resistance.

ANCOM‐bc2 analysis revealed that *Elizabethkingia anophelis* (padj = 5.0E‐03), *Cedecea neteri* (padj = 1.0E‐02), and *Chryseobacterium* sp. ZHDP1 (padj = 1.0E‐02) were significantly associated with the alive permethrin 1× phenotype, aligning with our previous findings. Furthermore, *Elizabethkingia anophelis* (padj = 3.0E‐02) and *Chryseobacterium* sp. ZHDP1 (padj = 3.0E‐02) exhibited significant associations with the alive 5× phenotype, reinforcing the potential role of these species in resistance escalation.

Intriguingly, the analysis also identified *Serratia marcescens* (padj = 5.0E‐02) as significantly associated with pyrethroid susceptibility (Table S10). These findings not only corroborate our earlier results but also offer a more comprehensive view of the microbial landscape associated with resistance across varying concentrations of permethrin.

This dual‐approach analysis underscores the consistency and significance of our results, providing a solid foundation for further exploration of microbial signatures in the context of pyrethroid resistance.

## 5 Discussion

This study used a comprehensive approach encompassing PoolSeq Genome‐wide association studies with three different designs, Sanger sequencing and combined genotyping to detect genomic and microbial signatures associated with pyrethroid resistance escalation in *An*. *funestus* population in Cameroon.

### 5.1 Temporal Genomic Identified Major Genetic Loci Associated With Pyrethroid Resistance Escalation in *Anopheles funestus* in Mibellon

The study reveals no significant genetic divergence between alive and dead *Anopheles funestus* phenotypes at 1×, 5× and 10× insecticide concentrations. However, resistance signals emerged at the *rp1* QTL on chromosome 2R and the *CYP9* locus on the X chromosome when comparing field phenotypes to the susceptible FANG strain. The absence of strong genomic differentiation in F_1_ field phenotypes challenges the identification of resistance‐linked variants, unlike in *Drosophila melanogaster* (Bastide et al. 2013). Similar studies using PoolSeq and GWAS in *An. funestus*, *An. gambiae*, and *An. coluzzii* also failed to detect genomic variations linked to pyrethroid resistance (Hearn et al. 2022). This may be due to shared genetic backgrounds between alive and dead phenotypes, intense resistance already established in the population where resistant haplotypes are fixed in both alive and dead, and mosquitoes' rapid evolutionary response to selection pressures.

To overcome these challenges, genetic crosses and temporal genomic analysis were employed (Cattel et al. 2020). These approaches identified strong differentiation on chromosome 2R and X (mean *F*_*ST*_ > 0.3), overlapping with the *rp1* QTL and *CYP9* loci containing major cytochrome P450 enzymes. Temporal comparisons between 2014 and 2021 unexposed *An. funestus* populations revealed major resistance‐associated peaks on 2R and X, confirming previous findings. The *rp1* QTL explains 87% of genetic variance in pyrethroid resistance (Wondji et al. 2007), while *CYP9K1*, previously overexpressed in Uganda, is linked to resistance across Cameroon (Djoko Tagne et al. 2024a; Gadji et al. 2024). The 2014 population lack major differentiation from FANG at *rp1* and *CYP9* loci, whereas the 2021 population exhibited strong peaks, confirming the temporal emergence and fixation of these resistance loci in Mibellon population.

Selective sweeps spanning *rp1* and *CYP9* loci were observed, driven by metabolic resistance genes. A valley of reduced genetic diversity in *rp1*, with negative Tajima's D values, indicated strong selection in field phenotypes, resistant genetic crosses, and the 2021 population compared to susceptible crosses and 2014 samples. Similar selective sweeps at *CYP6 rp1* were previously documented in Malawi between 2002 and 2014, but not in Cameroon at that time (Barnes et al. 2017). The absence of a sweep in 2014 aligns with the lack of widespread LLIN use, suggesting selection was driven by LLINs or agricultural pesticides.

The *CYP9K1* locus also exhibited a strong selective sweep, with drastically reduced genetic diversity over just 7 years. This suggests that resistance evolution in Cameroon extends beyond *rp1* to other genomic regions, highlighting the complexity of resistance mechanisms and the adaptability of *An. funestus* populations. These findings parallel previous cases of positive selection for target site mutations (e.g., kdr) in mosquito vectors (Odero et al. 2024; Riveron et al. 2014).

### 5.2 Point Mutations Are Associated With Pyrethroid Resistance Evolution in *Anopheles funestus* in Mibellon

Significant replacement polymorphisms at increasing allelic frequencies were identified in two genomic regions under positive selection. The strongest was *CYP9K1* (G454A), a major selective sweep reducing genetic diversity between 2014 and 2021 in *An. funestus* from Mibellon. Previously fixed in Uganda, this SNP was linked to *CYP9K1* overexpression and insecticide resistance across Cameroon (Djoko Tagne et al. 2024; Gadji et al. 2024). It was nearly fixed (> 90%) in 2021 samples (alive, dead and unexposed), but rare (5%) in 2014 samples and the FANG susceptible strain, with higher frequency in alive crosses than dead ones. Initially absent in Cameroon in 2014, it reached fixation in under 8 years, likely due to insecticide pressure (Hearn et al. 2022). Previous GWAS and functional genomics confirmed its spread, enabling the design of a DNA‐based resistance marker (Hearn et al. 2022b; Djoko Tagne et al. 2024).

Within the *CYP6 rp1* cluster, novel significant SNPs were found in multiple *CYP6* genes. Except for H169R, all *CYP6P4a* and *CYP6P4b* mutations were reported at low frequencies (< 15%) in *An. funestus* from the Democratic Republic of the Congo (Acford‐Palmer et al. 2023). The *GSTe2* L119F mutation, associated with permethrin and DDT resistance in Cameroon (Acford‐Palmer et al. 2023) and Benin (Riveron et al. 2014), and the novel K146T mutation, also identified in the DRC (Acford‐Palmer et al. 2023), were found at moderate frequencies in field populations but absent in FANG. No relevant knockdown mutations were detected in *An. funestus VGSC*, unlike *An. gambiae* (Martinez‐Torres et al. 1998). Overall, increasing allele frequencies of metabolic resistance genes likely drive resistance evolution in Mibellon.

### 5.3 Signature of Complex Genomic Anomalies Are Associated With Pyrethroid Resistance Evolution in *Anopheles funestus* in Mibellon

#### 5.3.1 Mobile Elements Associated With Resistance Evolution in *Anopheles funestus* in Mibellon

Analysis and visualisation of field samples identified a pattern characteristic of a 4.3 kb transposable element (TE) located downstream *CYP6P9b* and upstream *CYP6P5* genes in all the alive and dead field phenotypes. Similar pattern of TE's was detected in Ugandan *An. funestus* population in 2014 but not or at apparently very low frequency in Cameroon having lower coverage depth compared to Uganda 2014 population. Similarly, another TE was also identified upstream *CYP9K1* gene in *An*. *funestus* from Cameroon similar to previously identified in Uganda in 2014 (Weedall et al. 2020b) suggesting possible evidence of gene flow between Uganda and Cameroon mosquito populations. Unfortunately, our analyses were unsuccessful in assembling the complete inserted sequence of the transposable element (TE) located upstream of *CYP9K1*. Both mobile elements were absent in a fully susceptible strain (FANG) and exhibiting temporal changes between 2014 and 2021 suggesting their potential role in the dynamic of resistance this population. Similar findings of transposable element insertions in mosquito genomes have been reported in previous studies, underscoring the dynamic nature of mosquito genomes and their role in adaptation (Huddleston et al. 2017; Palatini et al. 2020). This observation also aligns with previous studies that highlight the role of triple mutant including a Zanzibar‐like transposable elements in adaptation to environmental stressors, including deltamethrin resistance (Njoroge et al. 2022).

#### 5.3.2 Duplications Associated With Resistance Evolution in *Anopheles funestus* in Mibellon

Gene duplications play a crucial role in resistance evolution by amplifying detoxification and resistance‐related genes. This study identified novel duplications, including a 2.5 kb region spanning partial *CYP6P4a/b* and a 5.8 kb region spanning *CYP9K1*. Additional duplications were detected: a 6.4 kb region covering *CYP6AA1* and partial *CYP6AA2*, and a 3.4 kb region spanning two carboxylesterases within *rp1*. Duplication patterns varied across regions, with different *CYP6AA1*, *CYP6AA2* and carboxylesterase duplications reported in Cameroon, Benin, and Ghana (Weedall et al. 2020b). The *CYP9K1* duplication, found in Uganda, resembled that of Cameroon but was absent in 2014. Similar duplications in *An. gambiae* have been linked to increased enzymatic activity and resistance (Faucon et al. 2015; Lucas et al. 2023). The identification of duplications in genes associated with resistance evolution in *An*. *funestus* underscores the dynamic nature of the genomic adaptations that occur in response to selective pressures, shedding light on potential mechanisms driving resistance in this important malaria vector.

#### 5.3.3 Synergistic Impact of Structural (4.3 Kb) and Metabolic Markers (G454A) is Associated With Increased Pyrethroid Resistance in *An. funestus* From Mibellon

The combined genotyping of the 4.3 kb structural variant (SV) and *CYP9K1* (G454A) in *Anopheles funestus* hybrids post‐exposure to pyrethroids strongly supports its role in pyrethroid resistance aggravation. The combination of a single copy of each resistance marker (SV+/SV− and RS+/RS−) drastically increases the probability of surviving insecticide exposure (OR: 126.6), suggesting an additive or even multiplicative effect of these genetic variations. This highlights the complexity of resistance evolution, where multiple mechanisms can interact to enhance resistance levels beyond the contribution of individual markers. The presence of both markers in the same mosquito likely provides a dual advantage by improving both metabolic detoxification and structural changes that enhance survival. This finding is consistent with observations in East Africa, where three distinct genomic variations, the duplication of *CYP6AA1*, the presence of a Zanzibar‐like transposable elements, and the *CYP6P4* (I236M) point mutation collectively explain deltamethrin resistance in *Anopheles gambiae* (Njoroge et al. 2022). These findings have significant implications for vector control strategies. The rapid spread of these resistance alleles could undermine the efficacy of pyrethroid‐based interventions such as insecticide‐treated nets (ITNs) and indoor residual spraying (IRS). Continuous monitoring of these markers and their combinations in natural populations is crucial for predicting resistance trends and guiding insecticide resistance management strategies. Future studies should investigate the functional mechanisms underlying the 4.3 kb structural variation, assess their impact on cross‐resistance and assess whether alternative insecticides or resistance‐breaking interventions can be developed to mitigate the spread of resistance in *An. funestus*.

### 5.4 Microbial Signature Analysis Showed Association Between Specific Bacteria Taxa and Resistance Evolution in *Anopheles funestus* From Mibellon

Metagenomic analysis found 99.34% bacterial presence in mosquitoes, with dominant *Proteobacteria*, *Bacteroidetes*, *Actinobacteria* and *Firmicutes*, aligning with existing literature (Lee et al. 2023). However, no significant associations were found within and between samples diversities, suggesting similar microbial diversity with minimal or no insecticide impact on the microbiota of this *An. funestus* population from Mibellon (Dada et al. 2019; Pelloquin et al. 2021). Nevertheless, specific bacteria genera such as *Elizabethkingia* and *Cedecea* exhibited a strong association with permethrin resistance evolution, consistent with previous findings (Ingham et al. 2021), suggesting its potential contribution to resistance escalation. The overabundance of *Elizabethkingia anophelis* in highly resistant phenotypes lays the groundwork for future research that may attempt to isolate these bacteria from mosquito microbiota and carried out in vitro insecticide degradation experiment to establish the causality link between the microbiota and resistant phenotype. Conversely, *Asaia*, *Serratia*, and *Delftia* were consistently associated with susceptibility to pyrethroids which aligns with previous reports in *An. gambiae* from Cote d'Ivoire (Pelloquin et al. 2021; Omoke et al. 2021).

These bacteria, previously not linked with insecticide susceptibility in *An. funestus*, present opportunities for further research, potentially leading to the development of new vector intervention tools. Future experimental studies should target *Asaia*, *Serratia*, and *Delftia* to understand their functional roles in susceptibility restoration and the dynamics of parasitic infection, providing insights into sustainable vector control strategies, including the potential utilisation of these bacteria as microbial‐based larvicides (Antonio‐Nkondjio et al. 2021).

## 6 Conclusion

Overall, this study provides strong evidence of the extensive selective sweeps spread across two different genomic regions and acting on cytochrome P450‐based metabolic resistance to insecticides in *Anopheles funestus* from Mibellon, Cameroon. We conclude that positive selection on these regions has rapidly occurred in Mibellon between 2014 and 2021 in response to the increased control intervention strategies such as LLINs. These two genomic regions are more complex than previously appreciated and provide a foundation for future studies aiming at the development of novel molecular markers for resistance evolution management. It is also important to explore alternative control strategies that do not rely on insecticides, such as paratransgenesis using candidate microbial symbionts identified in this study. This is crucial because resistance will inevitably emerge if we continue switching from one insecticide to another.

## Author Contributions

Conceptualization was led by C.S.W. Data curation was performed by M.G. and J.A.K.‐O., while formal analysis was conducted by M.G. Funding acquisition and project administration were handled by C.S.W. Investigation involved M.G., J.A.K.‐O. and C.S.W., and methodology was developed by M.G., J.A.K.‐O., M.T. and C.S.W. Resources were provided by M.T., M.J.W., L.M.J.M. and J.H. Supervision and validation were carried out by J.H., B.O. and C.S.W. Visualisation was performed by M.G. and J.H. The original draft was written by M.G., and review and editing were contributed by all co‐authors.

## Funding

This work was supported by Bill and Melinda Gates Foundation, INV‐006003. Wellcome Trust, 217188/Z/19/Z.

## Disclosure

*Benefit‐Sharing Statement*: Benefits Generated: We have ensured that all collaborators are recognised as co‐authors. The findings of this research have been disseminated to the participating communities and the wider scientific community. This work addresses a critical priority, malaria vector control. Furthermore, our group is dedicated to promoting international scientific collaborations and strengthening institutional research capacity.

## Ethics Statement

This work was reviewed and approved by the National Ethics Committee for Health Research (CNERSH) of Cameroon (ID: 2021/07/1372/CE/CNERSH/SP).

## Consent

The authors have nothing to report.

## Conflicts of Interest

The authors declare no conflicts of interest.

## Supporting information


**Figure S1:** Study site map.
**Figure S2:** Interpretation of read pair orientations for the detection of signature of complex genomic rearrangement. LR indicates normal reads: The reads are left and right respectively of the unsequenced part of the sequenced DNA fragment when aligned back to the reference genome. LL or RR implies inversion in sequenced DNA with respect to the reference genome. RL implies duplication or translocation with respect to the reference genome.
**Figure S3:** Correlation plot among crosses.
**Figure S4:** Correlation plot among field F_1_ phenotypes.
**Figure S5:** Pairwise *F*_*ST*_ genetic differentiation between dead 1×, 5× and alive 5×, 10× *An. funestus* pools from Mibellon.
**Figure S6:** Pairwise *F*_*ST*_ genetic differentiation between *An. funestus* field phenotypes and FANG reference laboratory fully susceptible strain.
**Figure S7:** Genome‐wide Tajima's D in *An. funestus* in Mibellon. This was computed in overlapping windows of 50 kb moving in steps of 25 kb.
**Figure S8:** Best non‐synonymous polymorphisms associated to super‐resistance in *An. funestus* in Mibellon. The heatmap presents the top 35 differential ns‐SNPs identified from frequency and *p* values‐based filtering (see method) from the *CYP6* and *CYP9* cluster. For each position, allele frequency variation between each population/phenotype are shown as a blue‐red colour scale (higher frequency been represented in red colour scale). The grey colour for the Mibellon 2022 population represents position where allele frequencies could not be called due to the very low coverage depth.
**Figure S9:** Temporal evolution of candidate variants allelic frequencies in *An. funestus* population between 2014 and 2021.
**Figure S10:** Polymorphism analysis of *CYP6P9b* gene in *An. funestus* genetic crosses. A and B represent sequence alignment showing presence of two keys point mutations located within the coding region of *CYP6P9b* gene; C and D display phylogenetic tree and haplotype network while E and F indicate the association of *CYP6P9b* mutant allele 392 T with pyrethroid resistance phenotype in *An. funestus* hybrids. Alive_HR and Dead_HS denote highly resistant and highly susceptible phenotypes, respectively.
**Figure S11:** (A) IGV screenshot of the alignment around the *CYP6* region showing a pattern characteristic of a transposon insertion of 4.3 kb located downstream *CYP6P9b* and upstream *CYP6P5*. The blue box indicates the transposon insertion for each sample. (B) IGV screenshot of the coverage track around the *CYP6* region showing the TE insertion sequence “CAAATGTACA” located in the intergenic region of *CYP6P9b* and *CYP6P5*. The TE inserted is framed by a blue. (C) IGV screenshot of the alignment around the *CYP9* region showing a pattern characteristic of a huge transposable element upstream *CYP9K1* gene shown by the blue rectangle box for each sample. (D) IGV screenshot of the coverage track around the *CYP9* region showing the TE inserted sequence “CAAATTTC” upstream *CYP9K1* gene shown by the blue rectangle box for each sample. The blue rectangle box represents the region where the transposon is inserted. This structural variant was absent in 2014, FUMOZ and FANG populations but present in all field populations. (E) IGV screenshot of the alignment around the *CYP6* region showing a 6.4 kb duplication spanning the entire *CYP6AA1* and partial *CYP6AA2*. The duplicated region is shown in the blue rectangle box for each sample. Multiple others duplicated regions are found but with unclear breakpoints. (F) IGV screenshot of the alignment around the *CYP6* region showing a 16.8 kb duplication spanning the entire *CYP6AA1*, *CYP6AA2*, the 2× Carboxylesterases, a cytochrome P450 *AFUN008357* and partial *CYP6P9a*. The blue rectangle box indicates the duplicated region for each sample. (G) IGV screenshot of the alignment around the CYP6 region showing a 6.5 kb deletion in in the intergenic region between *CYP6P9a* and *CYP6P9b*. This indel is characterised by a drop in coverage depth in all the field samples and FANG shown by the blue rectangle box but is an insertion in FUMOZ population which exhibited high coverage depth shown by the red rectangle box. (H) IGV screenshot of the alignment around the *CYP6* region showing a 6.5 kb deletion in in the intergenic region between *CYP6P9a* and *CYP6P9b*. Normal pair‐end reads are represented by grey horizontal bars connected with a light line. Anomalous reads are represented in red rectangles connected with a long red line indicating larger insert size than expected. This indel is characterised by absence of normal alignment reads in all the field samples and FANG shown by the blue rectangle box but presence in FUMOZ population which has good and normal aligned reads shown by the red rectangle box. (I) IGV screenshot of the alignment around the *CYP6* region showing 2.5 kb duplication spanning partial *CYP6P4a* and *CYP6P4b*. The blue rectangle box represents the duplicated regions for each sample. This duplication was found in 2014 and FANG populations but just on two supporting pair‐end reads compared to 2021 populations exhibiting the duplication on more than 12 pair‐end reads indicating it emergence over time. (J) IGV screenshot of the alignment around the *CYP6* region showing 3.4 kb duplication spanning partial 2× Carboxylesterases. The blue box is showing the duplicated regions for each sample. (K) IGV screenshot of the alignment around the *CYP9* region showing a 5.8 kb duplication spanning the entire *CYP9K1* gene but just present in one unique sample, the control unexposed 2021. Other samples indicate high coverage depth around the duplicated region which may be of greatest concern. The location of the inserted sequence is shown by the blue rectangle box.
**Table S1:** list of primers used for the *CYP6P9b* full length amplification for Sanger sequencing.
**Table S2:** Mapping statistics of PoolSeq GWAS data.
**Table S3:** Coverage statistics of PoolSeq GWAS data.
**Table S4:** Genome‐wide pairwise *F*_*ST*_ between different *An. funestus* population phenotypes.
**Table S5:** Genome‐wide Pairwise *F*_*ST*_ between different *An. funestus* genetic crosses.
**Table S6:** Polymorphism analysis of *CYP6P9b* in *An. funestus* in hybrid genetic crosses from Mibellon.
**Table S7:** Summary statistics of reads classification to microbial kraken2 database.
**Table S8:** Microbial composition of *An. funestus* at the level of Kingdom, Phyla, Family, Class and genus.

**Table S9:** Non‐synonymous polymophisms associated with *CYP6* and *CYP9* selective sweeps in *An. funestus* from Mibellon.

**Table S10:** Key bacteria species associated with insecticide resistance evolution in *An. funestus* from Mibellon.

## Data Availability

Data sets of the PoolSeq whole genome sequencing are available in European Nucleotide Archive under accessions PRJEB87435. These data will be made publicly available on the 25th December 2025 following the publication of the related manuscript. All analysis codes utilised in this study are accessible within the GitHub repository via https://github.com/Gadji‐M/PoolSeq_OMIcsTouch. All supplementary Information are available in Zenodo (Mahamat Gadji 2025).
