# Supplementary material for: PoolSeq Genome‐Wide Association Studies and Microbial Signature Analyses Identify Novel Candidates Associated With Pyrethroid Resistance Evolution in Anopheles funestus in Cameroon
Source: Mol Ecol. 2026 Jan 5;35(1):e70220. doi: 10.1111/mec.70220 (PMC12768894; doi:10.1111/mec.70220)
Supplement: Supplementary file 1 — Figure S1: Study site map. Figure S2: Interpretation of read pair orientations for the detection of signature of complex genomic rearrangement. LR indicates normal reads: The reads are left and right respectively of the unsequenced part of the sequenced DNA fragment when aligned back to the reference genome. LL or RR implies inversion in sequenced DNA with respect to the reference genome. RL implies duplication or translocation with respect to the reference genome. Figure S3: Correlation plot among crosses. Figure S4: Correlation plot among field F1 phenotypes. Figure S5: Pairwise F ST genetic differentiation between dead 1×, 5× and alive 5×, 10× An. funestus pools from Mibellon. Figure S6: Pairwise F ST genetic differentiation between An. funestus field phenotypes and FANG reference laboratory fully susceptible strain. Figure S7: Genome‐wide Tajima's D in An. funestus in Mibellon. This was computed in overlapping windows of 50 kb moving in steps of 25 kb. Figure S8: Best non‐synonymous polymorphisms associated to super‐resistance in An. funestus in Mibellon. The heatmap presents the top 35 differential ns‐SNPs identified from frequency and p values‐based filtering (see method) from the CYP6 and CYP9 cluster. For each position, allele frequency variation between each population/phenotype are shown as a blue‐red colour scale (higher frequency been represented in red colour scale). The grey colour for the Mibellon 2022 population represents position where allele frequencies could not be called due to the very low coverage depth. Figure S9: Temporal evolution of candidate variants allelic frequencies in An. funestus population between 2014 and 2021. Figure S10: Polymorphism analysis of CYP6P9b gene in An. funestus genetic crosses. A and B represent sequence alignment showing presence of two keys point mutations located within the coding region of CYP6P9b gene; C and D display phylogenetic tree and haplotype network while E and F indicate the association of CYP6P9b muta [file MEC-35-e70220-s002.pdf]

MOLECULAR ECOLOGY

Supplemental Information for:

PoolSeq Genome-wide Association Studies and Microbial Signature Analyses Identify Novel Candidates Associated with Pyrethroid Resistance Evolution in *Anopheles funestus* in Cameroon

Mahamat Gadji [1,2]*, Jonas A Kengne-Ouafo [1], Magellan Tchouakui [1], Murielle J. Wondji[1,4], Leon M.J. Mugenzi [5], Jack Hearn [3], Boyomo Onana [2], Charles S. Wondji [1,4]*

Table of Contents:

Supplementary figures

Supplementary figure 1. Study site map. 2

Supplementary figure 2. Interpretation of read pair orientations for the detection of signature of complex genomic rearrangement. 3

Supplementary figure 3. Correlation plot among crosses. 4

Supplementary figure 4. Correlation plot among field F_1 phenotypes. 4

Supplementary figure 5. Pairwise F_{ST} genetic differentiation between dead 1 x, 5 x and alive 5 x, 10 x *An. funestus* pools from Mibellon. 5

Supplementary figure 6. Pairwise F_{ST} genetic differentiation between *An. funestus* field phenotypes and FANG reference laboratory fully susceptible strain. 6

Supplementary figure 7. Genome-wide Tajima's D in *An. funestus* in Mibellon. This was computed in overlapping windows of 50kb moving in steps of 25kb. 6

Supplementary figure 8. Best non-synonymous polymorphisms associated to super-resistance in *An. funestus* in Mibellon. 7

Supplementary figure 9. Temporal evolution of candidate variants allelic frequencies in *An. funestus* population between 2014 and 2021 8

MOLECULAR ECOLOGY

Supplementary figure 10. Polymorphism analysis of *CYP6P9b* gene in *An. funestus* genetic crosses. 9
Supplementary figure 11. *CYP6* and *CYP9*-based structural variations visualisation in IGV. 10

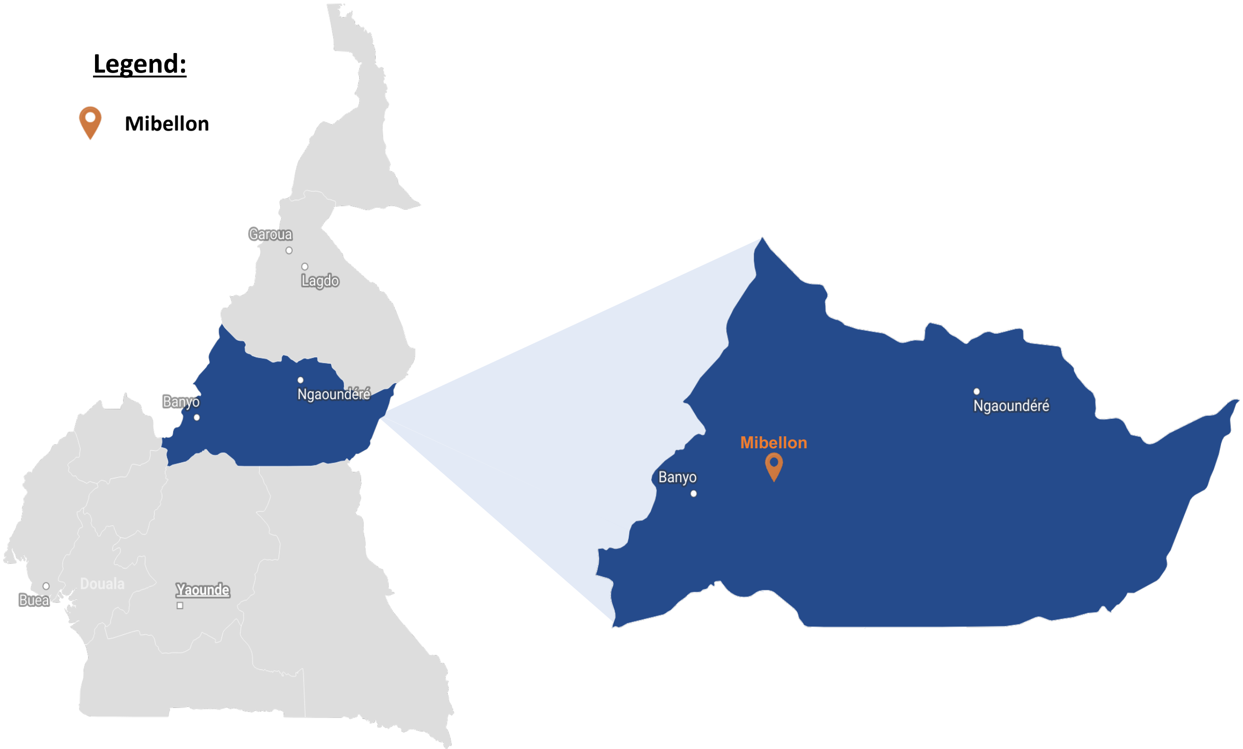

Supplementary figure 1. Study site map.

MOLECULAR ECOLOGY

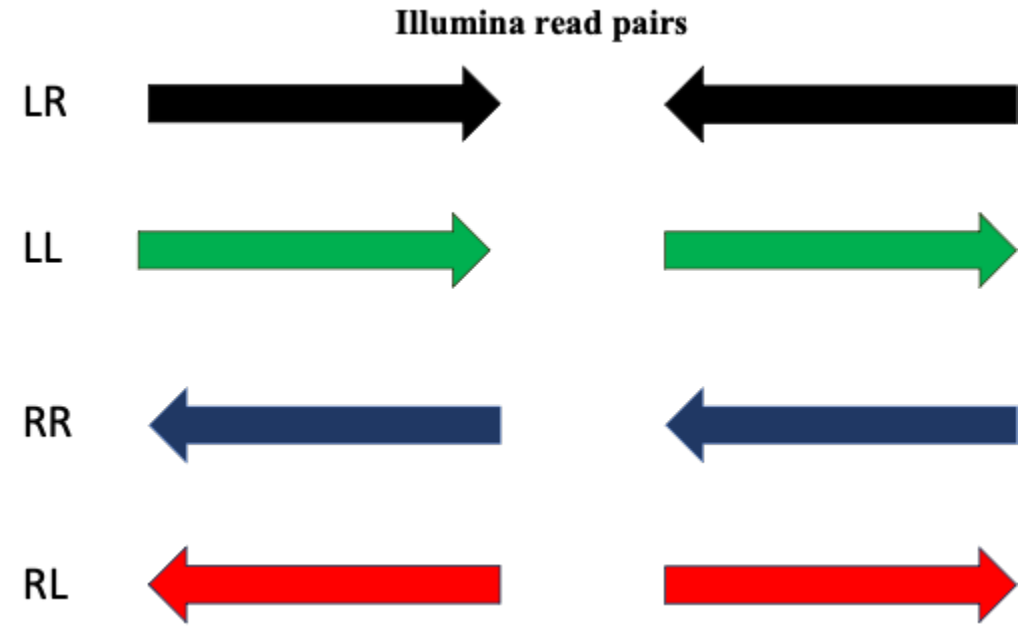

Supplementary figure 2. Interpretation of read pair orientations for the detection of signature of complex genomic rearrangement.

LR indicates normal reads: The reads are left and right respectively of the unsequenced part of the sequenced DNA fragment when aligned back to the reference genome. LL or RR implies inversion in sequenced DNA with respect to the reference genome. RL implies duplication or translocation with respect to the reference genome.

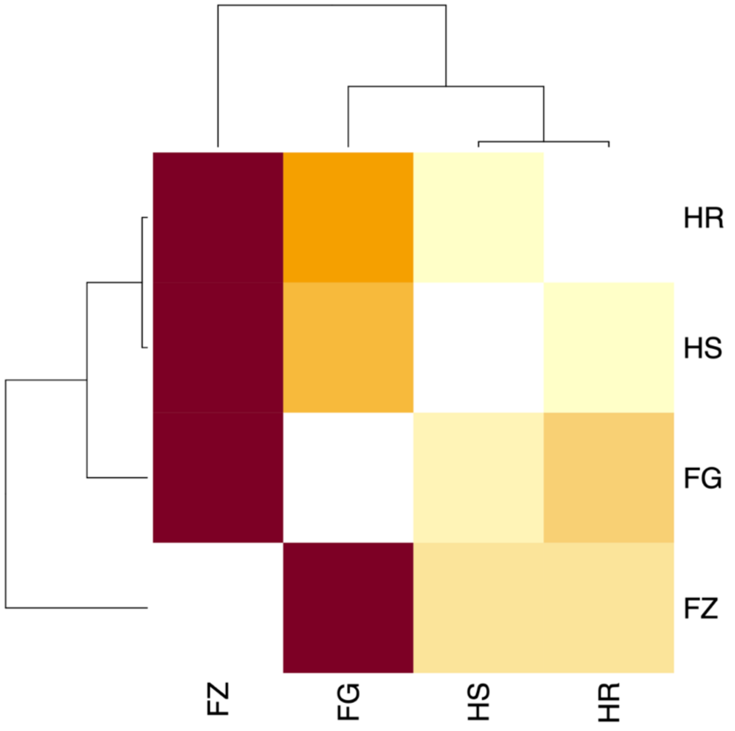

MOLECULAR ECOLOGY

Supplementary figure 3. Correlation plot among crosses.

The figure presents a correlation between genetics crosses and laboratory strains (FANG and FUMOZ). FZ, FG, HR and HS represent FUMOZ, FANG, Highly Resistant population and Highly Susceptible population, in that order.

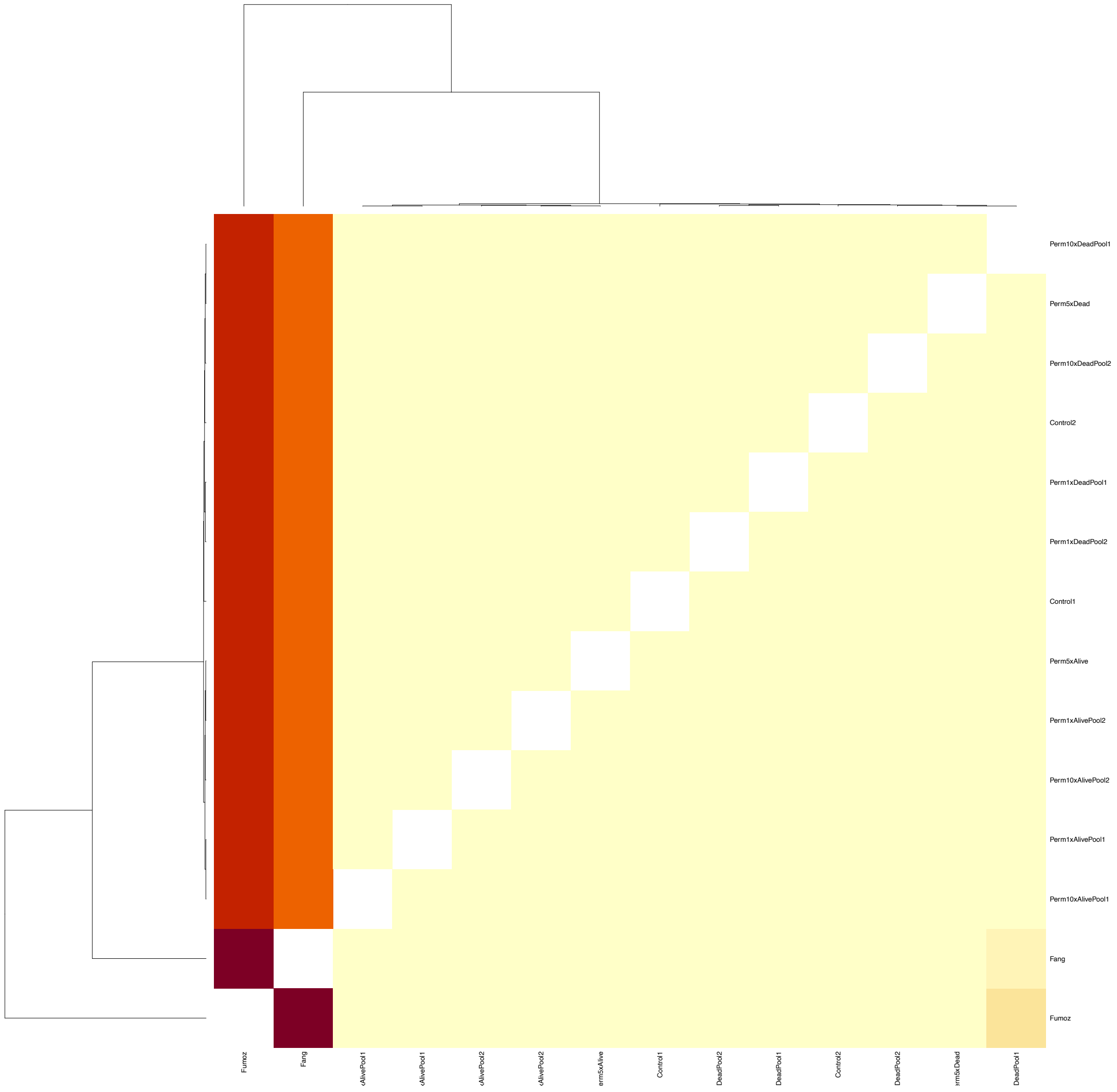

Supplementary figure 4. Correlation plot among field F_1 phenotypes.

MOLECULAR ECOLOGY

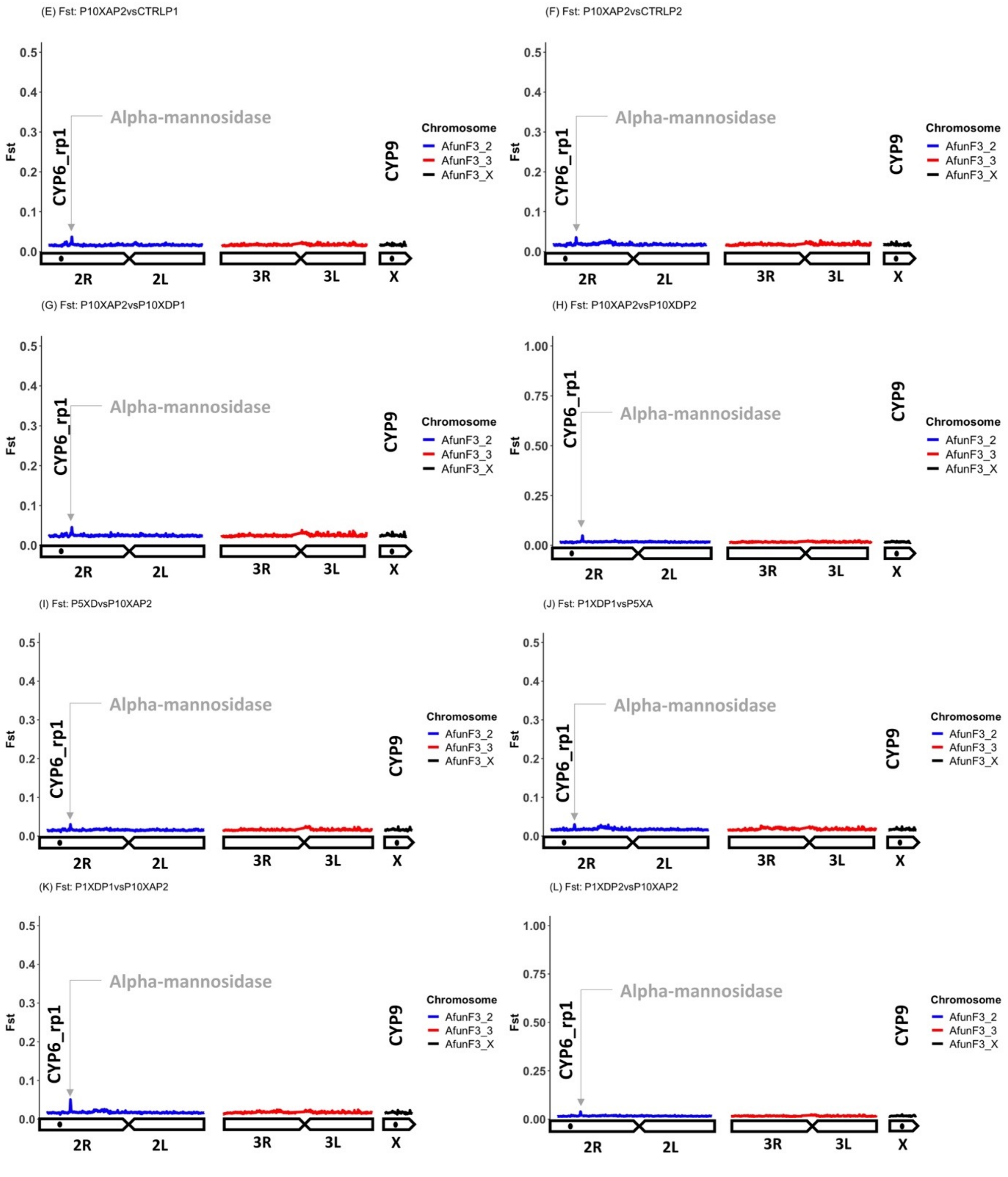

Supplementary figure 5. Pairwise F_{ST} genetic differentiation between dead 1 x, 5 x and alive 5 x, 10 x *An. funestus* pools from Mibellon.

MOLECULAR ECOLOGY

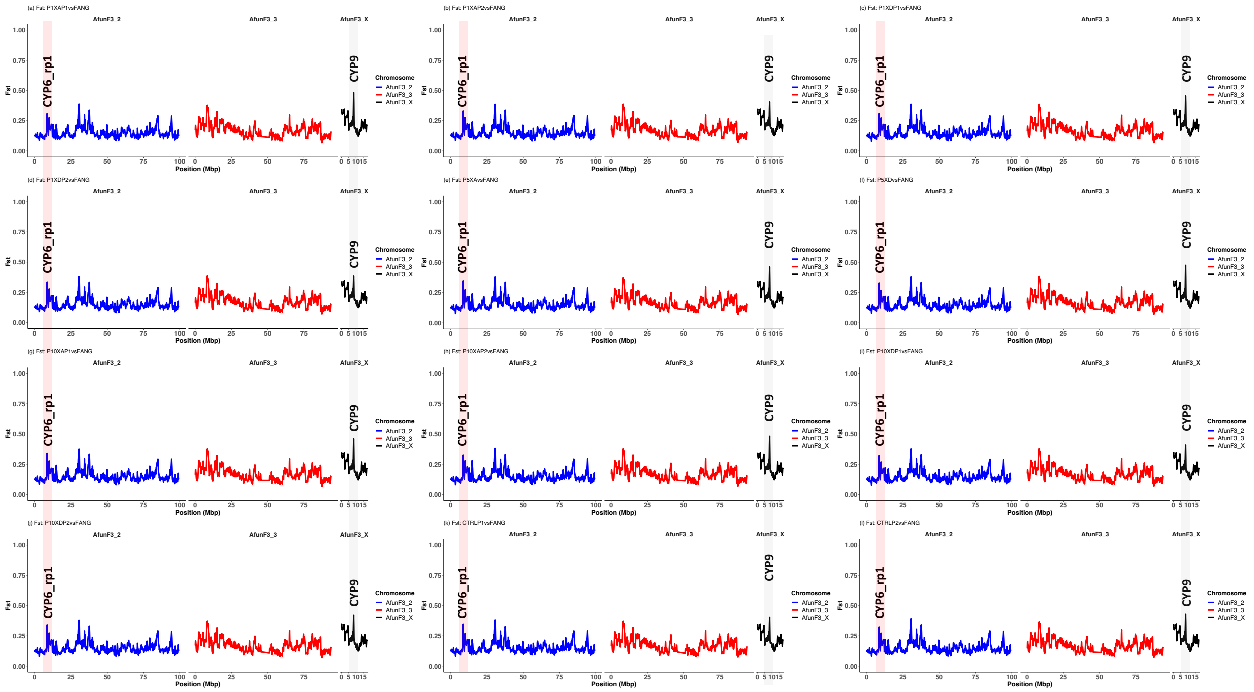

Supplementary figure 6. Pairwise F_{ST} genetic differentiation between *An. funestus* field phenotypes and FANG reference laboratory fully susceptible strain.

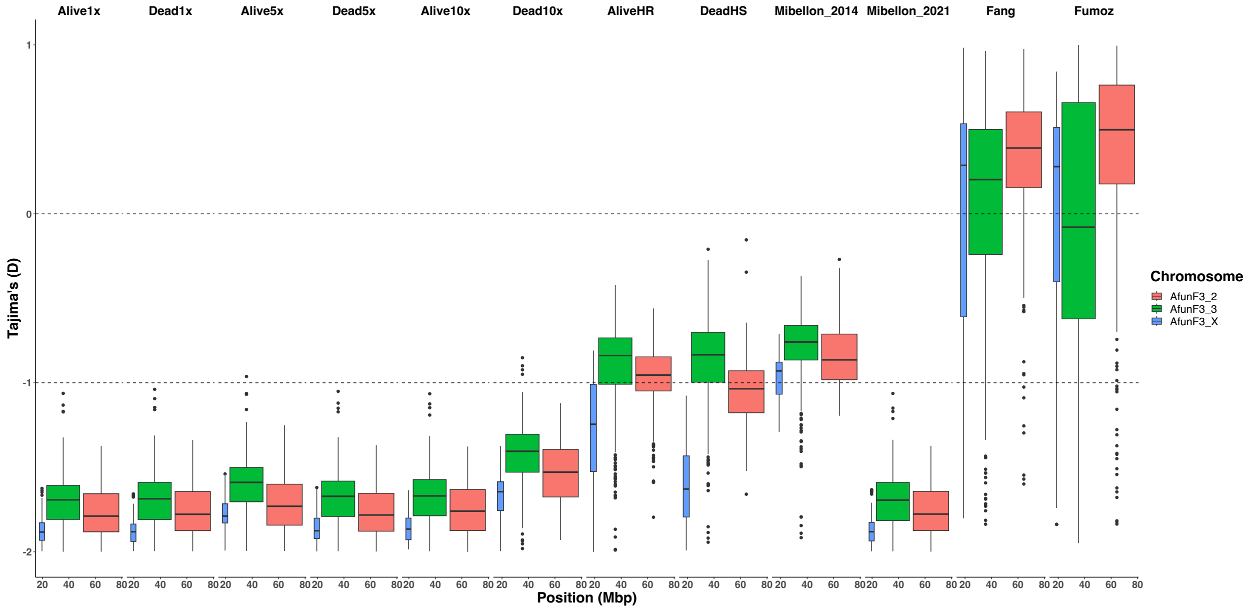

Supplementary figure 7. Genome-wide Tajima's D in *An. funestus* in Mibellon. This was computed in overlapping windows of 50kb moving in steps of 25kb.

MOLECULAR ECOLOGY

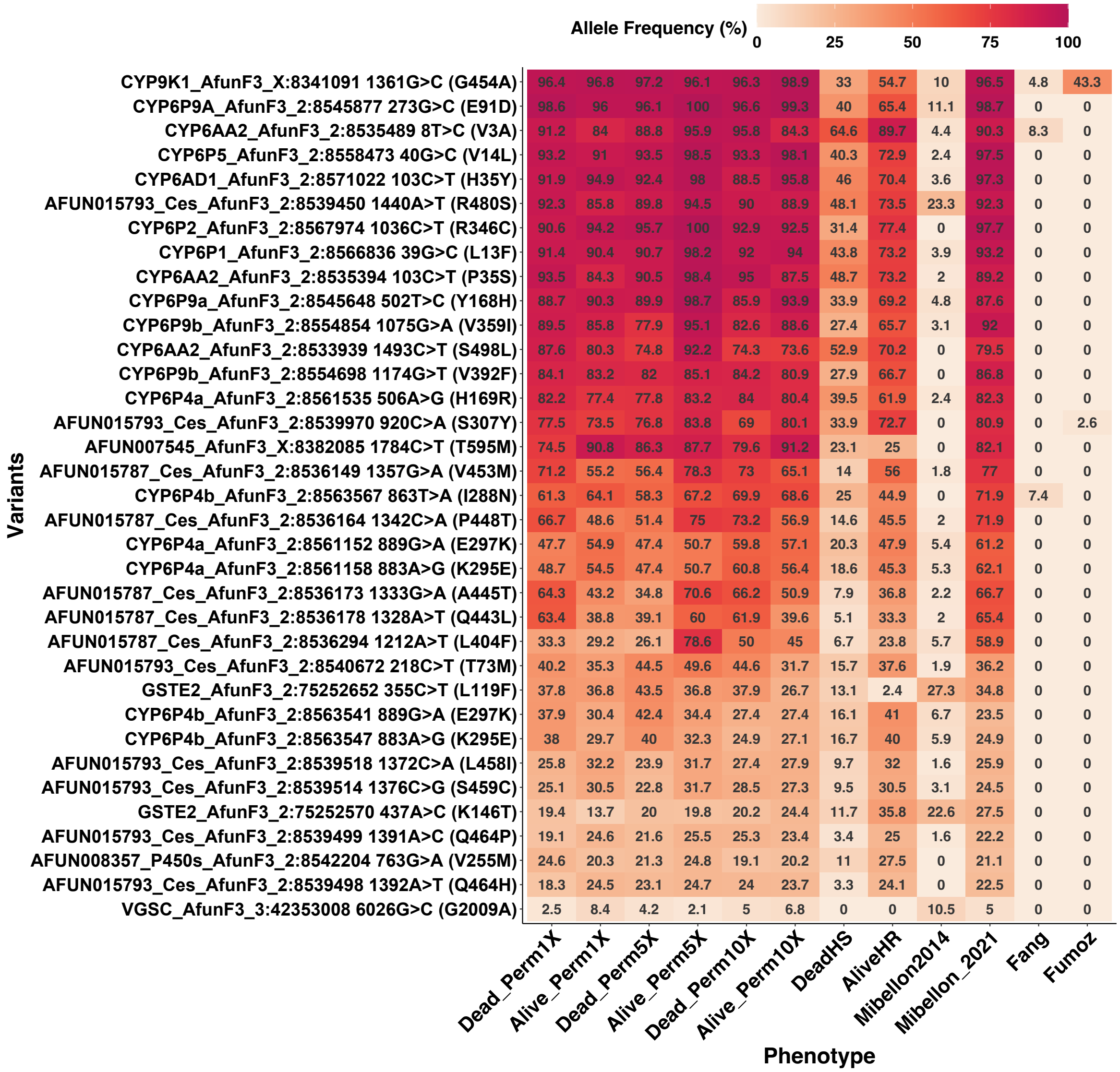

Supplementary figure 8. Best non-synonymous polymorphisms associated to super-resistance in *An. funestus* in Mibellon.

The heatmap presents the top 35 differential ns-SNPs identified from frequency and p values-based filtering (see method) from the *CYP6* and *CYP9* cluster. For each position, allele frequency variation between each population/phenotype are shown as a blue-red color scale (higher frequency been represented in red color scale). The grey color for the Mibellon 2022 population represents position where allele frequencies could not be called due to the very low coverage depth.

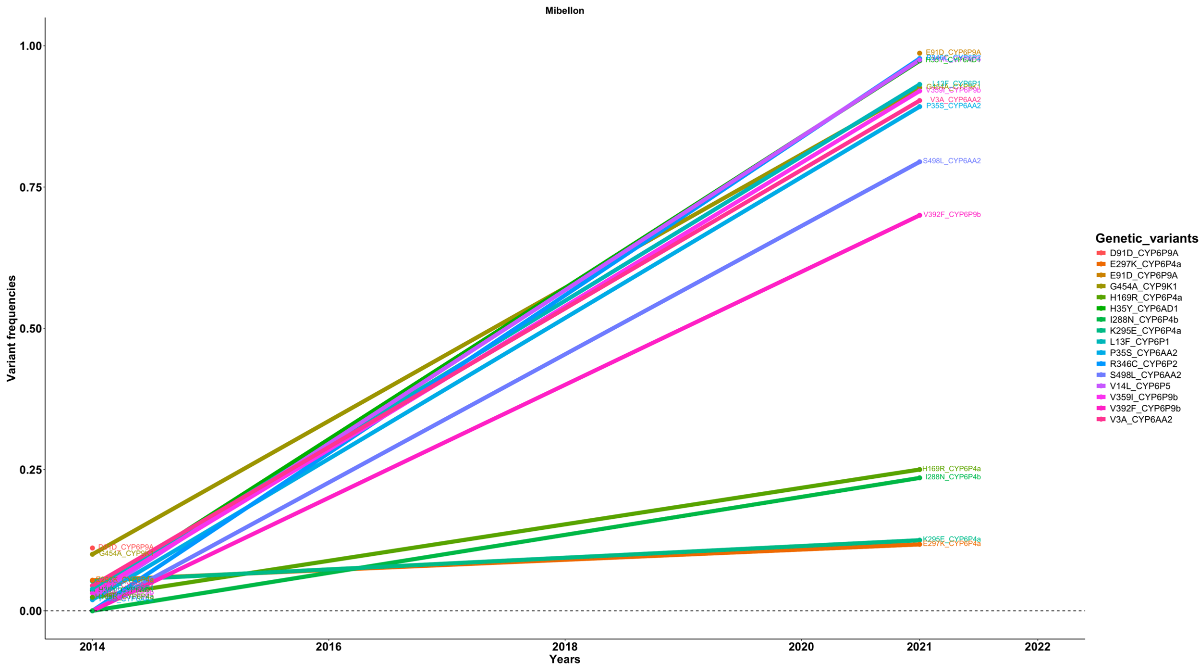

Supplementary figure 9. Temporal evolution of candidate variants allelic frequencies in *An. funestus* population between 2014 and 2021.

MOLECULAR ECOLOGY

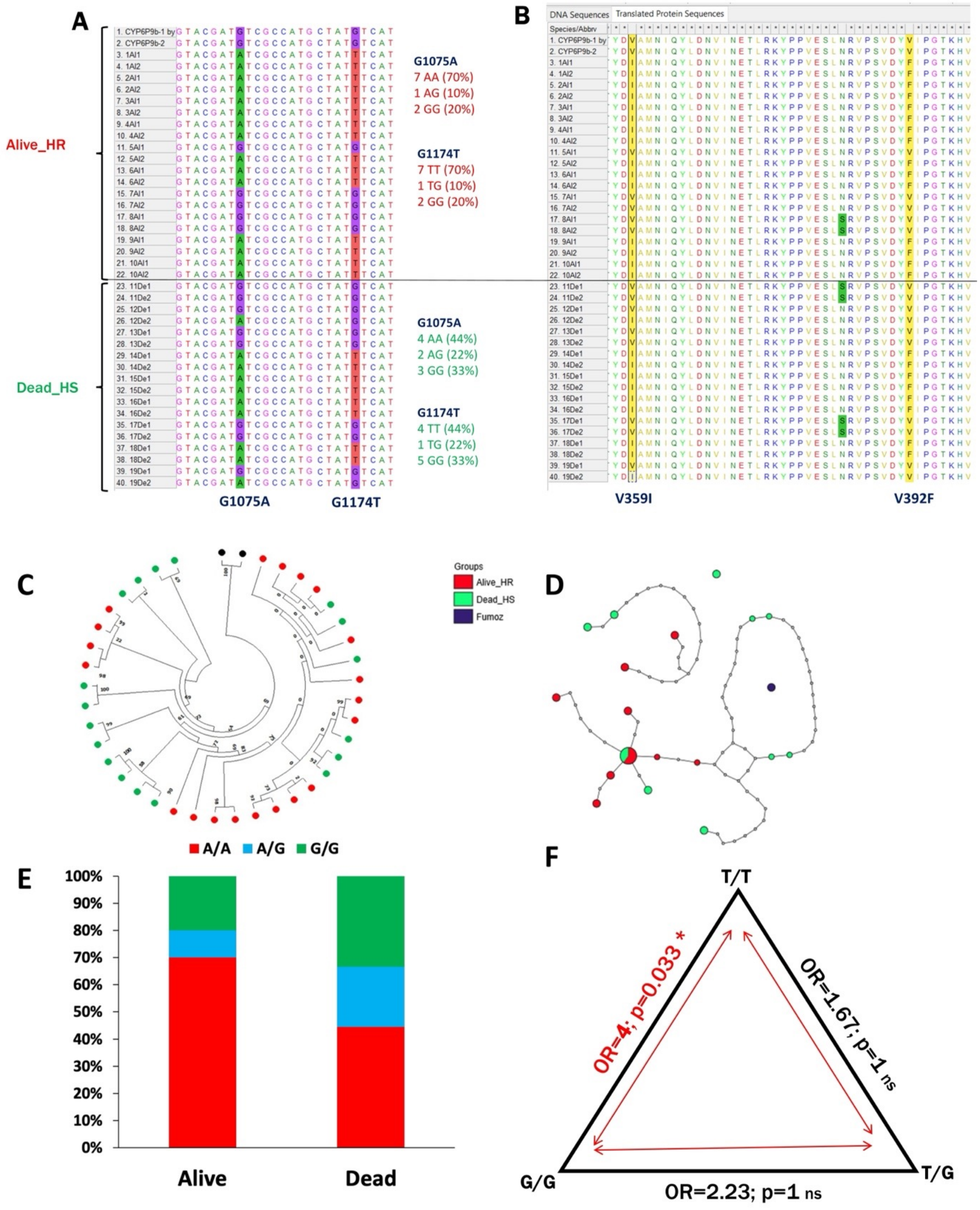

Supplementary figure 10. Polymorphism analysis of *CYP6P9b* gene in *An. funestus* genetic crosses.

MOLECULAR ECOLOGY

A and B represent sequence alignment showing presence of two keys point mutations located within the coding region of *CYP6P9b* gene; C and D display phylogenetic tree and haplotype network while E and F indicate the association of *CYP6P9b* mutant allele 392T with pyrethroid resistance phenotype in *An. funestus* hybrids. Alive_HR and Dead_HS denote highly resistant and highly susceptible phenotypes, respectively.

Supplementary figure 11. *CYP6* and *CYP9*-based structural variations visualisation in IGV.

Supplementary figure 11A. IGV screenshot of the alignment around the C*YP6* region showing a pattern characteristic of a transposon insertion of 4.3kb located downstream *CYP6P9b* and upstream *CYP6P5*.

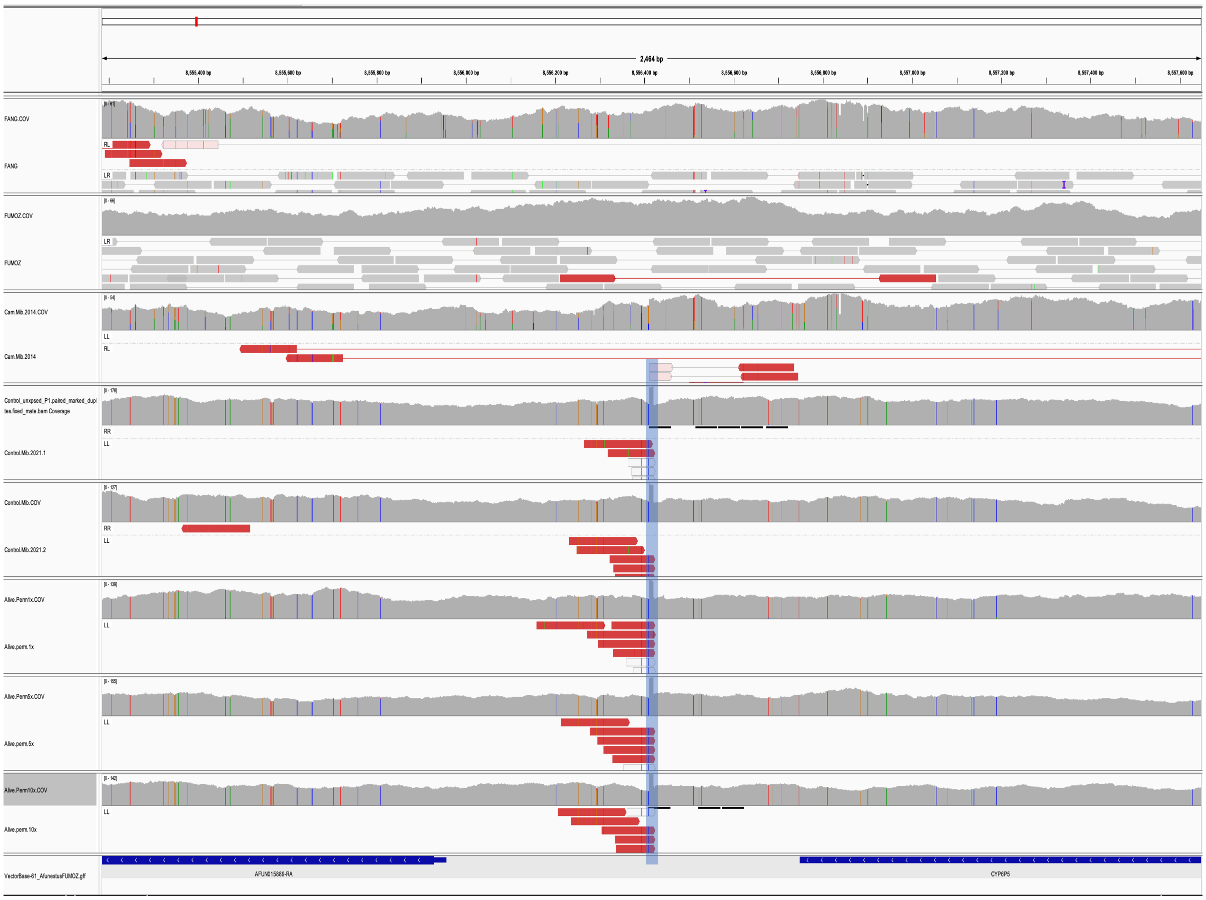

MOLECULAR ECOLOGY

Supplementary figure 11B. IGV screenshot of the coverage track around the *CYP6* region showing the TE insertion sequence "CAAATGTACA" located in the intergenic region of *CYP6P9b* and *CYP6P5.* The TE inserted is framed by a blue.

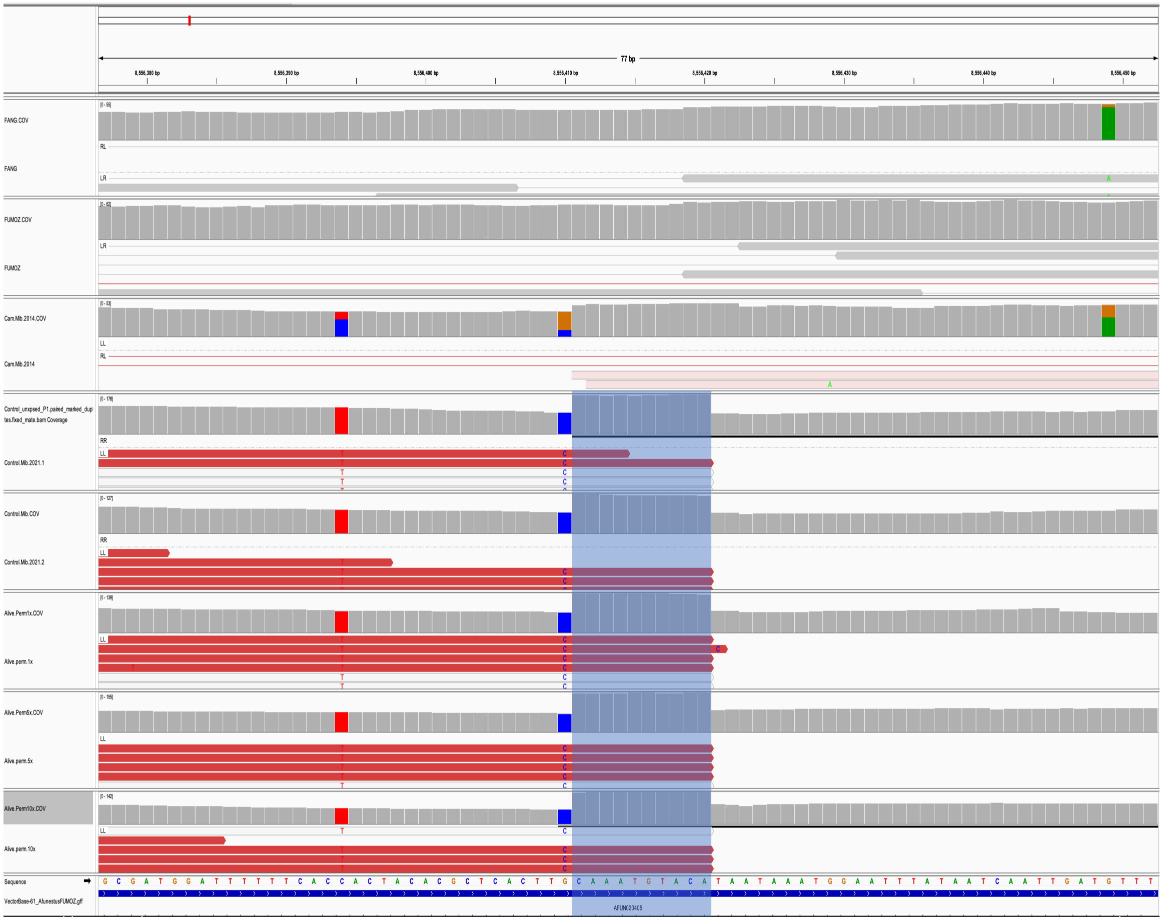

MOLECULAR ECOLOGY

Supplementary figure 11C. IGV screenshot of the alignment around the *CYP9* region showing a pattern characteristic of a huge transposable element upstream *CYP9K1* gene shown by the blue rectangle box for each sample.

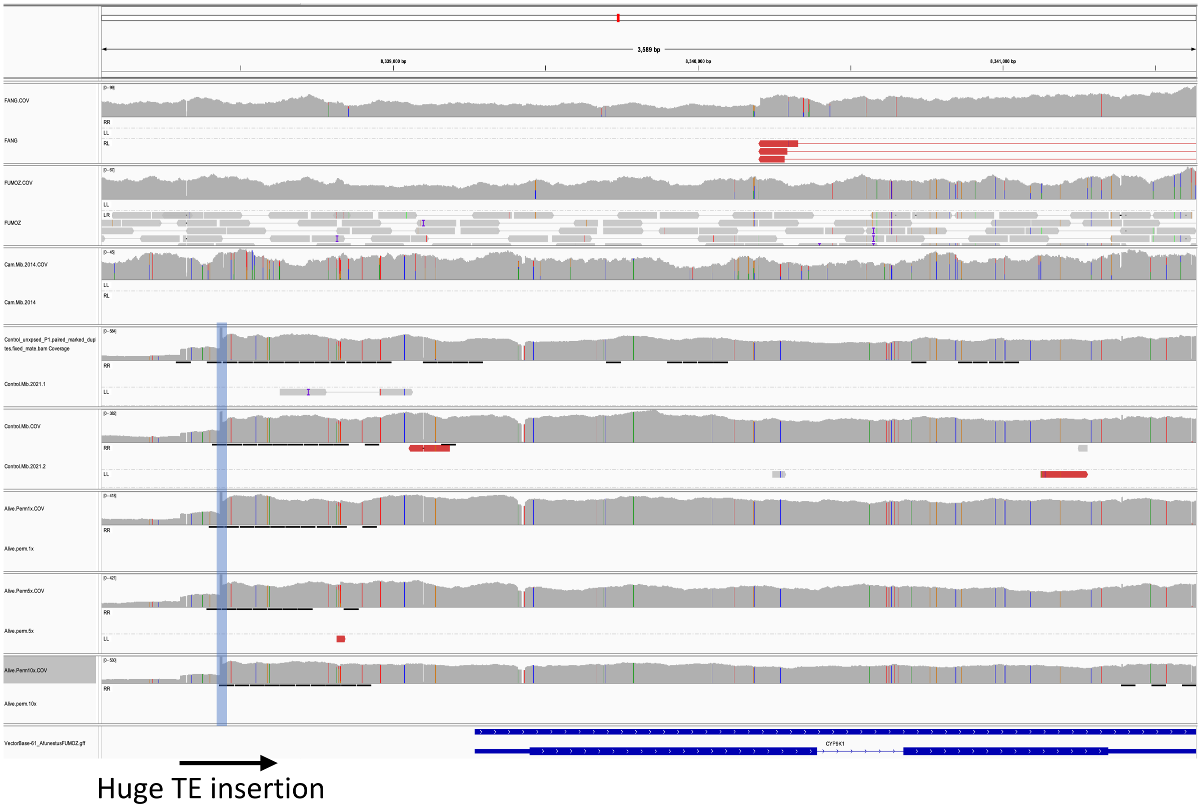

MOLECULAR ECOLOGY

Supplementary figure 11D. IGV screenshot of the coverage track around the *CYP9* region showing the TE inserted sequence “CAAATTTC” upstream *CYP9K1* gene shown by the blue rectangle box for each sample. The blue rectangle box represents the region where the transposon is inserted. This structural variant was absent in 2014, FUMOZ and FANG populations but present in all field populations.

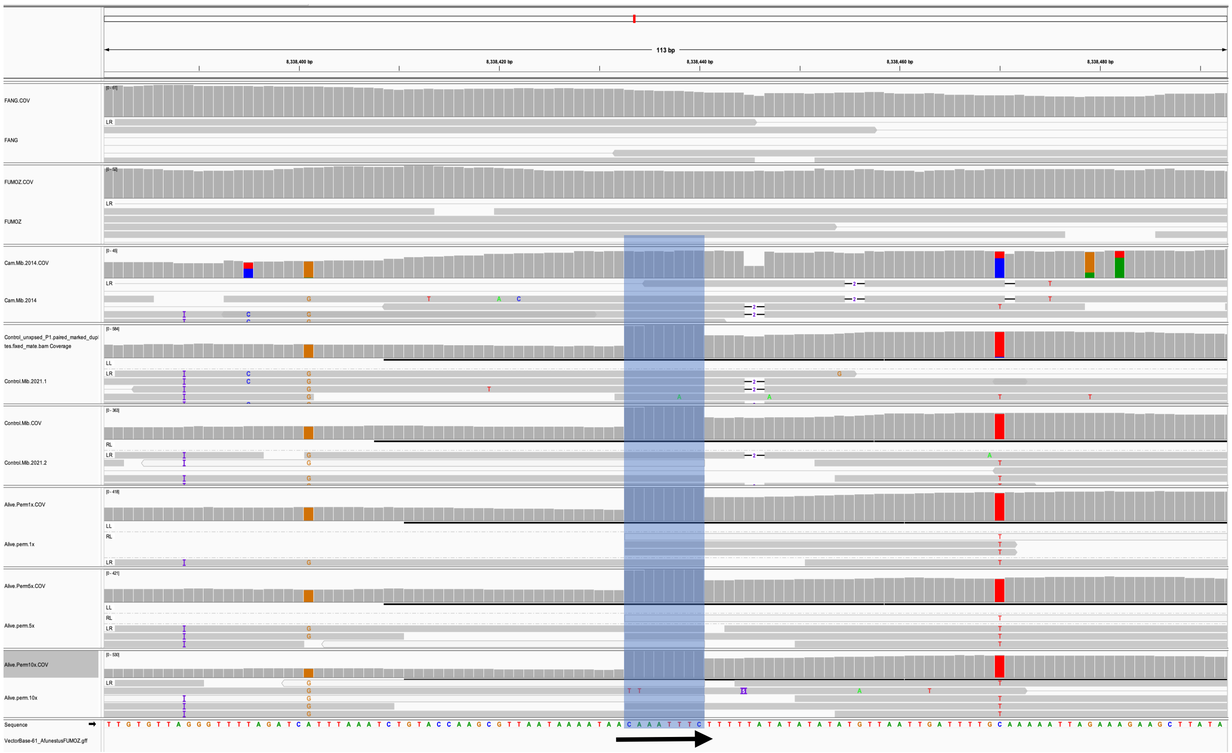

MOLECULAR ECOLOGY

Supplementary figure 11E. IGV screenshot of the alignment around the *CYP6* region showing a 6.4kb duplication spanning the entire *CYP6AA1* and partial *CYP6AA2*. The duplicated region is shown in the blue rectangle box for each sample. Multiple others duplicated regions are found but with unclear breakpoints.

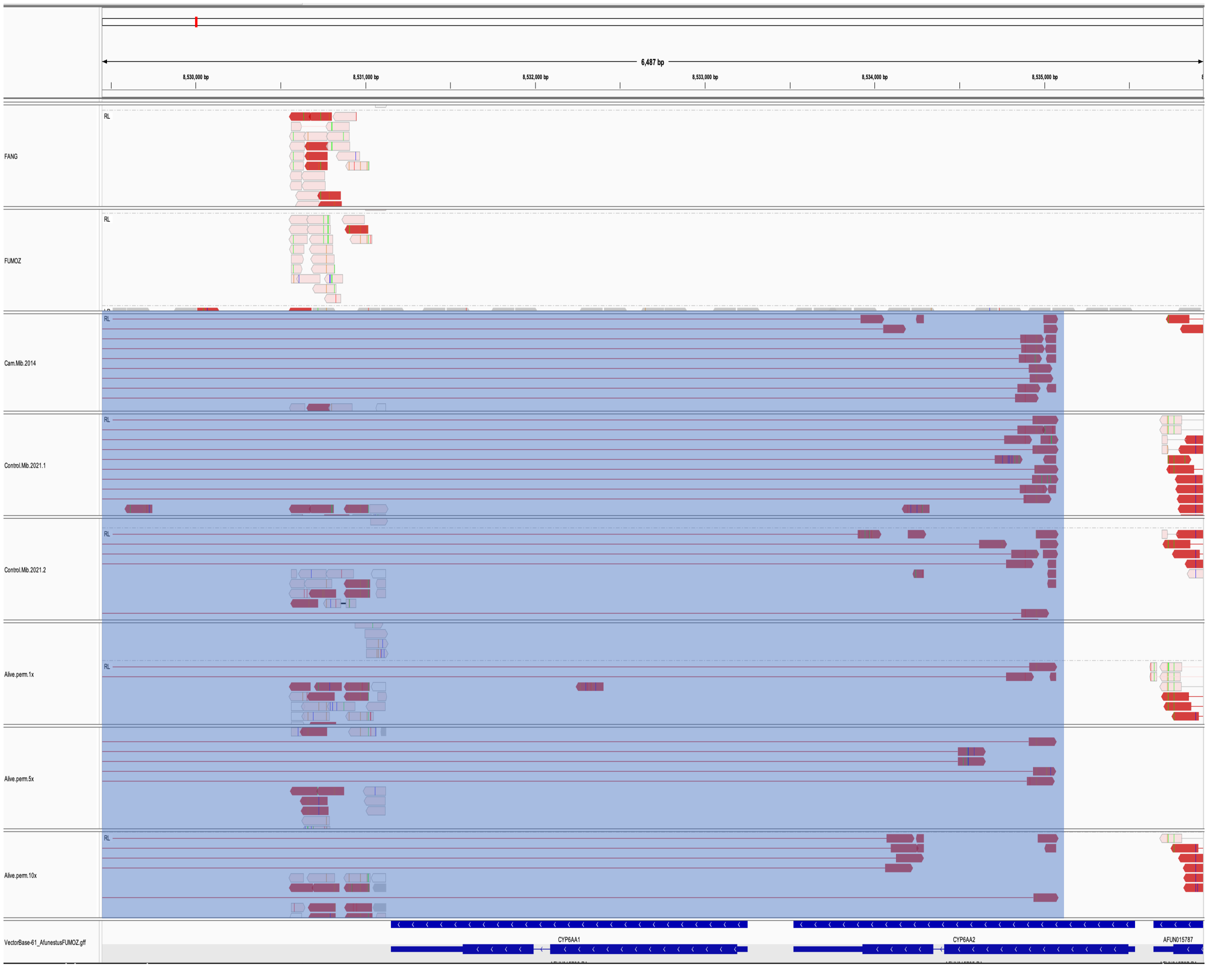

MOLECULAR ECOLOGY

Supplementary figure 11F. IGV screenshot of the alignment around the *CYP6* region showing a 16.8kb duplication spanning the entire *CYP6AA1*, *CYP6AA2*, the 2x Carboxylesterases, a cytochrome P450 *AFUN008357* and partial *CYP6P9a*. The blue rectangle box indicates the duplicated region for each sample.

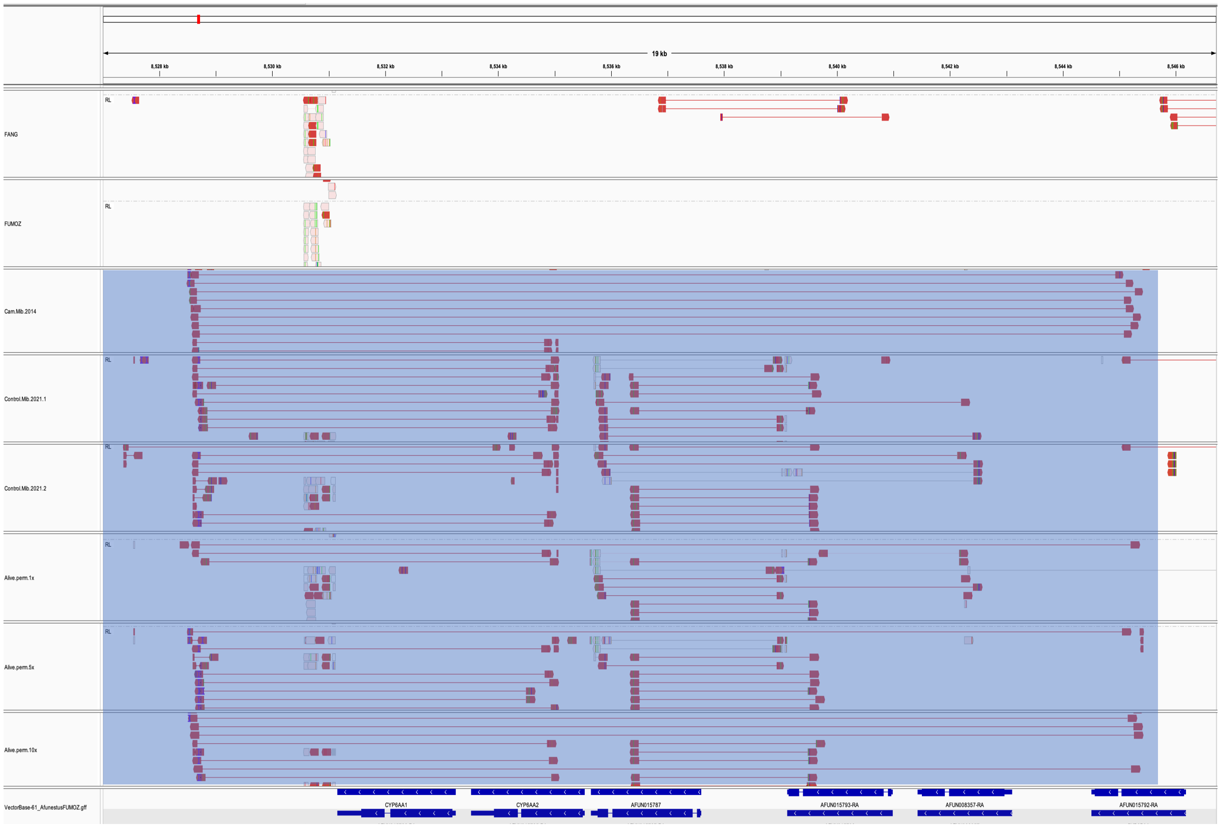

MOLECULAR ECOLOGY

Supplementary figure 11G. IGV screenshot of the alignment around the *CYP6* region showing a 6.5kb deletion in in the intergenic region between *CYP6P9a* and *CYP6P9b*. This indel is characterised by a drop in coverage depth in all the field samples and FANG shown by the blue rectangle box but is an insertion in FUMOZ population which exhibited high coverage depth shown by the red rectangle box.

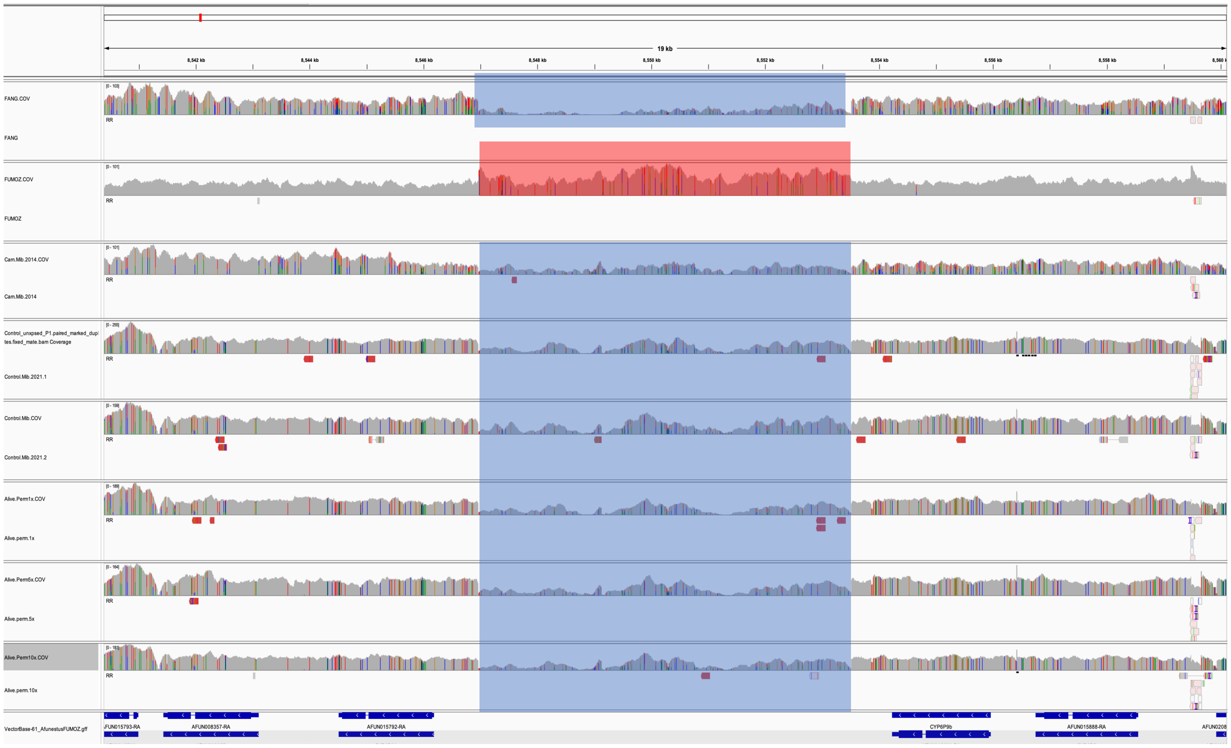

6.5 kb deletion

MOLECULAR ECOLOGY

Supplementary figure 11H. IGV screenshot of the alignment around the *CYP6* region showing a 6.5kb deletion in in the intergenic region between *CYP6P9a* and C*YP6P9b*. Normal pair-end reads are represented by grey horizontal bars connected with a light line. Anomalous reads are represented in red rectangles connected with a long red line indicating larger insert size than expected. This indel is characterized by absence of normal alignment reads in all the field samples and FANG shown by the blue rectangle box but presence in FUMOZ population which has good and normal aligned reads shown by the red rectangle box.

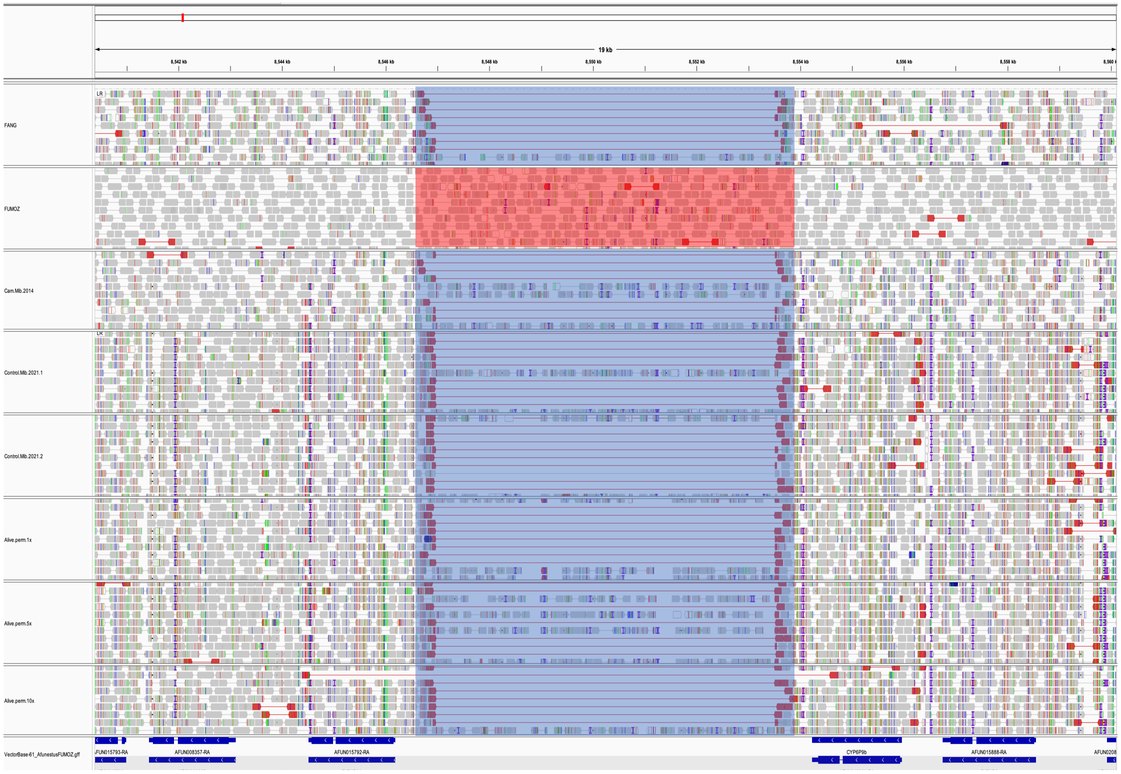

6.5 kb deletion

MOLECULAR ECOLOGY

Supplementary figure 11I. IGV screenshot of the alignment around the *CYP6* region showing 2.5kb duplication spanning partial *CYP6P4a* and *CYP6P4b*. The blue rectangle box represents the duplicated regions for each sample. This duplication was found in 2014 and FANG populations but just on two supporting pair-end reads compared to 2021 populations exhibiting the duplication on more than 12 pair-end reads indicating it emergence over time.

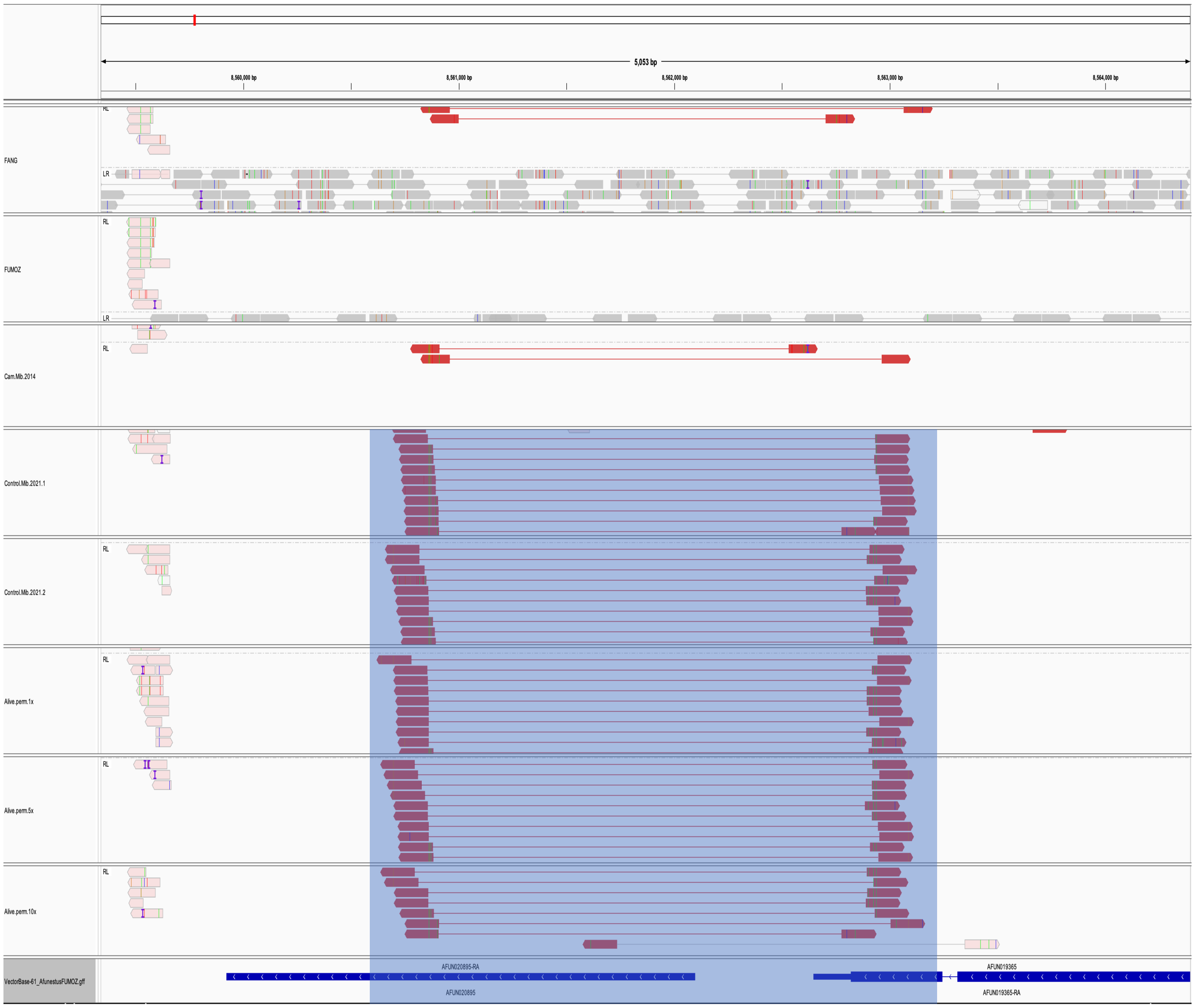

MOLECULAR ECOLOGY

Supplementary figure 11.J. IGV screenshot of the alignment around the *CYP6* region showing 3.4kb duplication spanning partial 2x Carboxylesterases. The blue box is showing the duplicated regions for each sample.

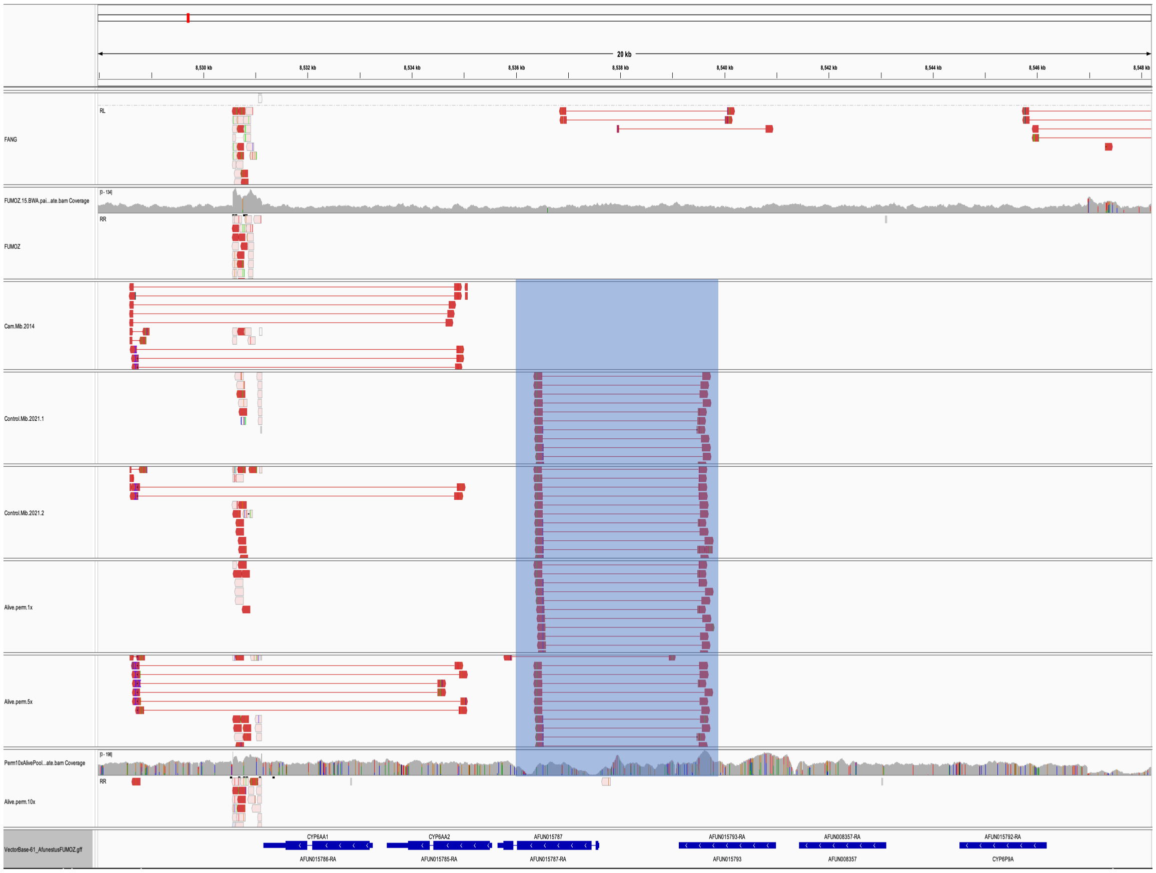

MOLECULAR ECOLOGY

Supplementary figure 11K. IGV screenshot of the alignment around the *CYP9* region showing a 5.8kb duplication spanning the entire *CYP9K1* gene but just present in one unique sample, the control unexposed 2021. Other samples indicate high coverage depth around the duplicated region which may be of greatest concern. The location of the inserted sequence is shown by the blue rectangle box.

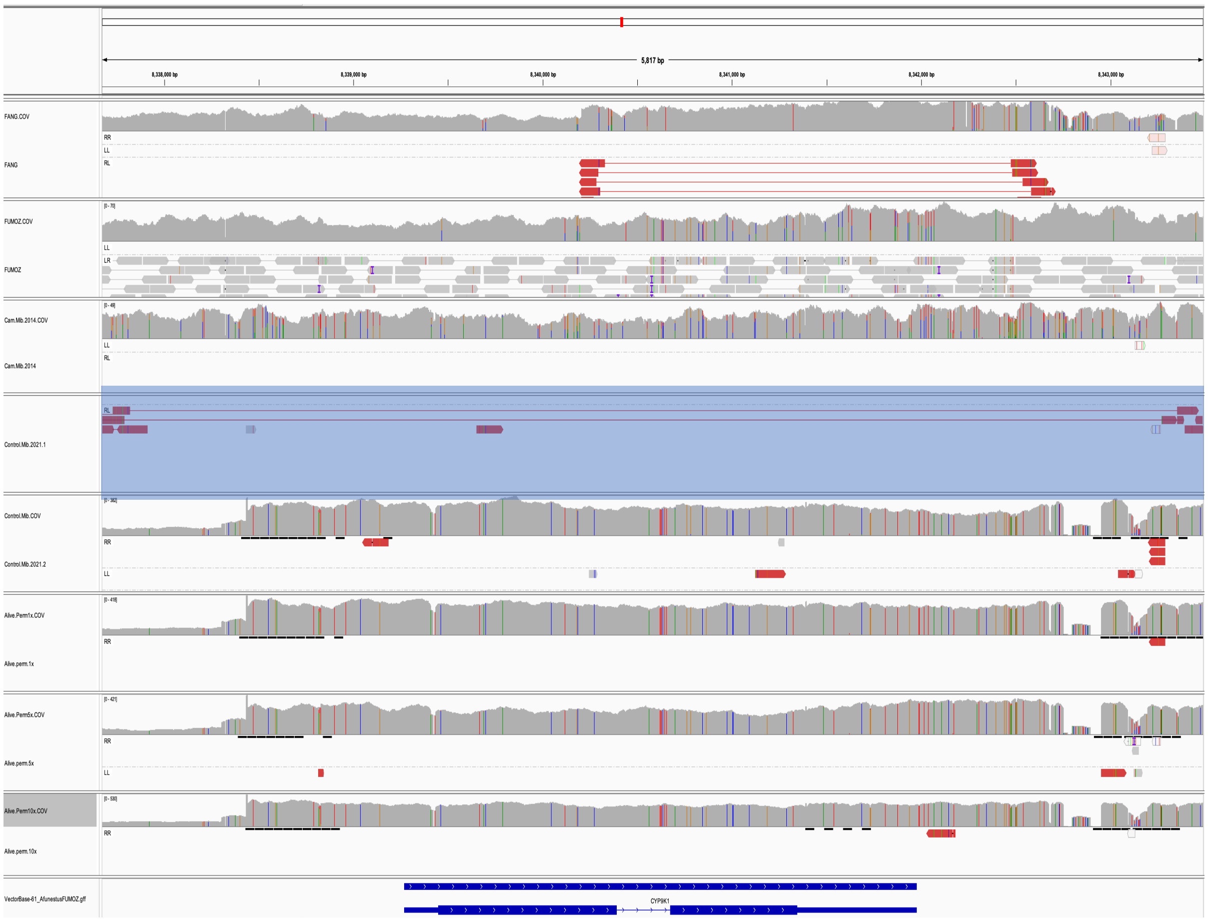

MOLECULAR ECOLOGY

Table of contents:

Supplementary tables

Supplementary table 1. list of primers used for full length *CYP6P9b* amplification. 21

Supplementary table 2. Mapping statistics of PoolSeq GWAS data. 22

Supplementary table 3. Coverage statistics of PoolSeq GWAS data. 22

Supplementary table 4. Pairwise F_{ST} between different *An. funestus* population phenotypes. 23

Supplementary table 5. Pairwise F_{ST} between different *An. funestus* genetic crosses. 24

Supplementary table 6. Polymorphism analysis of *CYP6P9b* in *An. funestus* hybrid genetic crosses from Mibellon, Cameroon. 25

Supplementary table 7. Summary statistics of reads classification to microbial kraken2 database. 25

Supplementary table 8. Microbial composition of *An. funestus* at the level of Kingdom, Phyla, Family, Class and genus. 26

Supplementary table 1. list of primers used for full length *CYP6P9b* amplification.

Primer name	Sequence
Mg_5'_6P9b_Fwd	TGACACGATGTGCTGATTACTC
Mg_5'_6P9b_Intnal_Fwd	GCGAGACGGTGAACTATC
Mg_5'_6P9b_Intnal_Rvse	GATAGTTCACCGTCTCGC

Mg_5'_6P9b_Rvse	GTTATCGTTGCACGGTAACT

Supplementary table 2. Mapping statistics of PoolSeq GWAS data.

Sample	R1	R2	R1/R2	Reads_aligned (%)	Aligned_in_pairs (%)	Singleton (%)
Alive_HR	80944407	80944407	161888814	149227150 (92)	148489446 (99)	8920456 (0.06)
Dead_HS	85757745	85757745	171515490	155900363 (91)	155171054 (99)	9821119 (0.06)
Perm1xAliveP1	88110592	88110592	176221184	164452974 (93)	163663262 (99)	9526800 (0.058)
Perm1xAliveP2	117342669	117342669	234685338	218664327 (93)	217642610 (99)	12860799 (0.06)
Perm1xDeadP1	90110933	90110933	180221866	162363855 (90)	161507546 (99)	10360051 (0.06)
Perm1xDeadP2	115775155	115775155	231550310	207777913 (89)	206820372 (99)	12862521 (0.06)
Perm5xAlive	82267980	82267980	164535960	151001713 (92)	150241046 (99)	9432983 (0.06)
Perm5xDead	93590590	93590590	187181180	173405036 (92)	172542262 (99)	11275792 (0.06)
Perm10xDeadP1	52000000	52000000	104000000	95363050 (92)	94908500 (99)	6183426 (0.06)
Perm10xDeadP2	105316705	105316705	210633410	191996883 (91)	191142184 (99)	11651939 (0.06)
Perm10xAliveP1	92064669	92064669	184129338	170846083 (93)	169973984 (99)	10448019 (0.06)
Perm10xAliveP2	95157677	95157677	190315354	173326745 (91)	172506242 (99)	10126311 (0.06)
ControlP1	136708527	136708527	273417054	227493943 (83)	226240138 (99)	15342953 (0.07)
ControlP2	87627676	87627676	175255352	156650142 (89)	155830786 (99)	9985670 (0.06)

Supplementary table 3. Coverage statistics of PoolSeq GWAS data.

Sample_ID	Chromosome	coverage	meandepth	meanbaseq	meanmapq

FgXMib_F2_HR	2_3_X	98.77	77.08	35.60	46.97
FgXMib_F2_HS	2_3_X	98.78	76.76	35.80	46.57
Cmr_Mib_2014	2_3_X	98.65	35.39	36.86	47.26
Perm1x_Al_P1	2_3_X	98.90	84.10	35.60	46.73
Perm1x_Al_P2	2_3_X	98.96	112.58	35.80	47
Perm1x_De_P1	2_3_X	98.91	83.83	35.53	46.30
Perm1x_De_P2	2_3_X	98.94	102.28	35.80	46.57
Perm5x_Al_P1	2_3_X	98.89	79.23	35.50	46.60
Perm5x_De_P1	2_3_X	98.93	91.17	35.60	46.37
Perm10x_Al_P1	2_3_X	98.93	92.30	35.60	47.40
Perm10x_Al_P2	2_3_X	98.92	90.66	35.63	47.03
Perm10x_De_P1	2_3_X	98.74	51.24	35.80	46.17
Perm10x_De_P2	2_3_X	98.94	97.46	35.77	46.07
Ctrl_unxp_P1	2_3_X	99.00	113.31	35.70	46.17
Ctrl_unxp_P2	2_3_X	98.91	80.25	35.50	46.47

Supplementary table 4. Pairwise F_{ST} between different *An. funestus* population phenotypes.

**Phenotypes**	**Alive1x _1**	**Alive1x _2**	**Dead1x_ 1**	**Dead1x_2**	**Alive5x**	**Dead5x**	**Alive10x _1**	**Alive10x _2**	**Dead10x _1**	**Dead10x _2**	**Ctrl_1**	**Ctrl_2**	**Fang**	**Fumoz**
**Alive1x _1**		0.001	0.002	0.002	0.002	0.002	0.001	0.003	0.003	0.004	0.005	0.003	**0.258**	**0.307**
**Alive1x _2**			0.003	0.002	0.001	0.003	0.003	0.002	0.004	0.005	0.006	0.004	**0.264**	**0.316**

MOLECULAR ECOLOGY

**Dead1x_1**	0.002	0.005	0.002	0.003	0.005	0.003	0.004	0.004	0.004	**0.257**	**0.306**
**Dead1x_2**		0.004	0.002	0.003	0.005	0.003	0.004	0.005	0.004	**0.250**	**0.296**
**Alive5x**			0.003	0.002	0.002	0.004	0.005	0.006	0.006	**0.270**	**0.324**
**Dead5x**				0.003	0.003	0.001	0.002	0.004	0.003	**0.272**	**0.327**
**Alive10x_1**					0.001	0.003	0.004	0.005	0.004	**0.255**	**0.304**
**Alive10x_2**						0.004	0.005	0.006	0.005	**0.257**	**0.306**
**Dead10x_1**							0.002	0.004	0.002	**0.270**	**0.324**
**Dead10x_2**								0.004	0.003	**0.252**	**0.299**
**Control_1**									0.006	**0.262**	**0.312**
**Control_2**										**0.260**	**0.309**
**Fang**											**0.457**
**Fumoz**											

Supplementary table 5. Pairwise F_{ST} between different *An. funestus* genetic crosses.

**Phenotypes**	**Alive_HR**	**Dead_HS**	**Fang**	**Fumoz**
**Alive_HR**	NA	0.036	**0.210**	**0.363**

**Dead_HS**	0.036	NA	**0.153**	**0.366**
**Fang**	**0.210**	**0.153**	NA	**0.438**
**Fumoz**	**0.363**	**0.366**	**0.438**	NA

Supplementary table 6. Polymorphism analysis of *CYP6P9b* in *An. funestus* hybrid genetic crosses from Mibellon, Cameroon.

Sample	N	S	Sys	Nsys	h	Hd	π	D	FuLi D
HR	20	44	29	15	9	0.889	0.0079	-0.099 ns	1.70
HS	18	62	39	24	10	0.928	0.0139	0.884 ns	1.71
HR_HS	38	76	45	32	18	0.920	0.0119	-0.165	1.97

Supplementary table 7. Summary statistics of reads classification to microbial kraken2 database.

Sample	Tot_raw_reads	Classified (%)	Chordate (%)	Artificial (%)	Unclassified (%)	Microbial (%)	Bacterial (%)	Viral (%)	Fungal (%)	Protozoan (%)
Ctrl_unx1	45619361	29357690 (64.35)	158497 (0.34)	0 (0)	16261671 (35.65)	29169769 (63.94)	29158088 (63.92)	4109 (0.009)	0 (0)	0 (0)

MOLECULAR ECOLOGY

Ctrl_unx2	18426108	7073700 (38.39)	126245 (0.68)	0 (0)	11352408 (61.61)	6929389 (37.61)	6897595 (37.43)	23807 (0.129)	0 (0)	0 (0)
Perm1x_Alive1	11638505	1707367 (14.67)	199100 (1.71)	0 (0)	9931138 (85.33)	1504716 (12.93)	1499226 (12.88)	799 (0.007)	0 (0)	0 (0)
Perm1x_Alive2	15841219	3521503 (22.23)	355874 (2.25)	0 (0)	12319716 (77.77)	3140906 (19.83)	3131019 (19.77)	3036 (0.019)	0 (0)	0 (0)
Perm1x_Dead2	17677979	6055943 (34.26)	140559 (0.79)	0 (0)	11622036 (65.74)	5908832 (33.42)	5901400 (33.38)	1760 (0.009)	0 (0)	0 (0)
Perm1x_Dead2	23649545	9055584 (38.29)	169056 (0.71)	0 (0)	14593961 (61.71)	8882027 (37.56)	8874110 (37.52)	1360 (0.006)	0 (0)	0 (0)
Perm5x_Alive	13408357	2804328 (20.91)	159041 (1.19)	0 (0)	10604029 (79.09)	2644733 (19.72)	2639527 (19.69)	904 (0.007)	0 (0)	0 (0)
Perm5x_Dead	13667681	1306802 (9.561)	319609 (2.34)	0 (0)	12360879 (90.44)	980422 (7.173)	973120 (7.12)	470 (0.003)	0 (0)	0 (0)
Perm10x_Alive1	16844063	5373280 (31.9)	141752 (0.84)	0 (0)	11470783 (68.1)	5226229 (31.03)	5219009 (30.98)	2550 (0.015)	0 (0)	0 (0)
Perm10x_Alive2	13115224	1568250 (11.96)	145505 (1.11)	0 (0)	11546974 (88.04)	1414432 (10.78)	1407948 (10.74)	1371 (0.010)	0 (0)	0 (0)
Perm10x_Dead1	33731524	5724864 (16.97)	451998 (1.34)	0 (0)	28006660 (83.03)	5264859 (15.61)	5248661 (15.56)	971 (0.003)	0 (0)	0 (0)
Perm10x_Dead2	18523892	3606005 (19.47)	245131 (1.32)	0 (0)	14917887 (80.53)	3360236 (18.14)	3354055 (18.11)	546 (0.003)	0 (0)	0 (0)

Supplementary table 8. Microbial composition of *An. funestus* at the level of Kingdom, Phyla, Family, Class and genus.

MOLECULAR ECOLOGY

Taxa	Frequency	Percentage
**Kingdom**		
Bacteria	2717	99.34
Viruses	12	0.44
Archaea	6	0.22
**Phylum**		
Proteobacteria	1535	56.12
Actinobacteria	915	33.46
Firmicutes	153	5.59
Bacteroidetes	89	3.25
Heunggongvirae	11	0.4
Planctomycetes	10	0.37
Euryarchaeota	6	0.22
Deinococcus	5	0.18
Fusobacteria	4	0.15
Cyanobacteria	3	0.11
Acidobacteria	2	0.07
Bamfordvirae	1	0.04
Gemmatimonadetes	1	0.04
**Class**		
Actinobacteria	915	33.46
Gammaproteobacteria	717	26.22
Alphaproteobacteria	518	18.94
Betaproteobacteria	283	10.35
Bacilli	134	4.9
Flavobacteriia	61	2.23
other classes	Other	3.9
**Order**		

Hyphomicrobiales	291	10.64
Micrococcales	280	10.24
Pseudomonadales	264	9.65
Enterobacterales	254	9.29
Burkholderiales	245	8.96
Corynebacteriales	239	8.74
Streptomycetales	143	5.23
Propionibacteriales	135	4.94
Bacillales	84	3.07
Sphingomonadales	81	2.96
Xanthomonadales	73	2.67
Flavobacteriales	61	2.23
Moraxellales	58	2.12
Rhodospirillales	57	2.08
Rhodobacterales	54	1.97
Lactobacillales	50	1.83
Aeromonadales	37	1.35
Micromonosporales	37	1.35
Caulobacterales	31	1.13
other Order (54)	261	9.6
Family		
Acetobacteraceae	263	9.62
Acidiferrobacteraceae	143	5.23
Actinomycetaceae	114	4.17
Actinopolymorphaceae	113	4.13
Actinopolysporaceae	97	3.55
Aerococcaceae	85	3.11
Aeromonadaceae	76	2.78

Alcaligenaceae	74	2.71
Alteromonadaceae	72	2.63
Amorphaceae	70	2.56
Anaplasmataceae	70	2.56
Aurantimonadaceae	69	2.52
Azonexaceae	66	2.41
Azospirillaceae	62	2.27
Bacillaceae	58	2.12
Bacteroidaceae	58	2.12
Baekduiaceae	52	1.9
Beijerinckiaceae	52	1.9
Beutenbergiaceae	51	1.86
Bifidobacteriaceae	50	1.83
Bogoriellaceae	43	1.57
Boseaceae	42	1.54
Breoghaniaceae	39	1.43
Brevibacteriaceae	37	1.35
Brucellaceae	37	1.35
Bruguierivoracaceae	34	1.24
Budviciaceae	31	1.13
Burkholderiaceae	28	1.02
Other family (749)	749	27.54
**Genus**		
Abiotrophia	258	9.43
Acetobacter	136	4.97
Achromobacter	76	2.78
Acidibrevibacterium	62	2.27
Acidihalobacter	56	2.05

Acidipropionibacterium	51	1.86
Acidovorax	46	1.68
Acinetobacter	46	1.68
Actinoalloteichus	45	1.65
Actinocatenispora	44	1.61
Actinokineospora	42	1.54
Actinomadura	40	1.46
Actinomarinicola	38	1.39
Actinomyces	37	1.35
Actinoplanes	37	1.35
Actinopolymorpha	34	1.24
Actinopolyspora	32	1.17
Actinosynnema	31	1.13
Actinotalea	31	1.13
Acuticoccus	26	0.95
Aerococcus	25	0.91
Aeromicrobium	23	0.84
Aeromonas	23	0.84
Aerosticca	22	0.8
Afipia	21	0.77
Aggregatibacter	21	0.77
Agrobacterium	20	0.73
Agrococcus	20	0.73
Agromyces	18	0.66
Ahniella	17	0.62
Alcaligenes	17	0.62
Alicycliphilus	16	0.59
Alloactinosynnema	16	0.59

Allobranchiibius	15	0.55
Allokutzneria	15	0.55
Allosaccharopolyspora	13	0.48
Altererythrobacter	13	0.48
Alteromonas	13	0.48
Aminobacter	12	0.44
Amycolatopsis	12	0.44
Anaerococcus	12	0.44
Anaeromyxobacter	12	0.44
Ancylobacter	11	0.4
Anoxybacillus	11	0.4
Aquabacter	11	0.4
Aquabacterium	11	0.4
Aquaspirillum	11	0.4
Aquibium	11	0.4
Aquihabitans	10	0.37
Aquincola	10	0.37
Aquisphaera	10	0.37
Aquitalea	10	0.37
Arachnia	10	0.37
Archangium	10	0.37
Arenimonas	10	0.37
Aromatoleum	10	0.37
Arsenicicoccus	9	0.33
Arthrobacter	9	0.33
Asaia	9	0.33
Asticcacaulis	9	0.33
Atlantibacter	9	0.33

Aurantimonas	8	0.29
Auraticoccus	8	0.29
Aureimonas	8	0.29
Auritidibacter	8	0.29
Austwickia	8	0.29
Azoarcus	8	0.29
Azospira	8	0.29
Azospirillum	8	0.29
Azotobacter	8	0.29
Bacillus	8	0.29
Bifidobacterium	7	0.26
Blastococcus	7	0.26
Bordetella	7	0.26
Bosea	7	0.26
Boudabousia	7	0.26
Brachybacterium	7	0.26
Bradyrhizobium	7	0.26
Brenneria	7	0.26
Breoghania	7	0.26
Brevibacterium	6	0.22
Brevilactibacter	6	0.22
Brevundimonas	6	0.22
Brucella	6	0.22
Buchnera	6	0.22
Burkholderia	6	0.22
Buttiauxella	6	0.22
Caballeronia	6	0.22
Campylobacter	6	0.22

Capnocytophaga	6	0.22
Other Genera (797)	797	30
